# A Multilevel Person-Centered Examination of Teachers' Workplace Experiences: Replication and Extension With Links to Instructional Support and Achievement

**DOI:** 10.3389/fpsyg.2021.711173

**Published:** 2021-08-06

**Authors:** Rebecca J. Collie, Andrew J. Martin, Alexandre J. S. Morin, Lars-Erik Malmberg, Pamela Sammons

**Affiliations:** ^1^School of Education, University of New South Wales, Sydney, NSW, Australia; ^2^Substantive Methodological Synergy Research Laboratory, Department of Psychology, Concordia University, Montreal, QC, Canada; ^3^Department of Education, University of Oxford, Oxford, United Kingdom

**Keywords:** job demands-resources theory, teacher well-being, latent profile analysis, multilevel, student achievement

## Abstract

In a replication and extension of an earlier study, we relied on person-centered analyses to identify teacher (Level 1) and school (Level 2) profiles based on teachers' experiences of job demands (barriers to professional development, disruptive student behavior), job resources (teacher collaboration, input in decision-making), and personal resources (self-efficacy). We examined data from 5,439 teachers working in 364 schools in Australia and 2,216 teachers working in 149 schools in England. Latent profile analysis revealed six teacher profiles: Low-Demand-Flourisher (11%), Mixed-Demand-Flourisher (17%), Job-Resourced-Average (11%), Balanced-Average (14%), Mixed-Resourced-Struggler (11%), and Low-Resourced-Struggler (36%). Two school profiles were identified: an Unsupportive school profile (43%) and a Supportive school profile (57%). Several significant relations between these profiles and teacher/school characteristics and work-related outcomes were also identified at both levels. Although our results generally replicated prior findings, some differences were also observed, possibly as a results of recent changes in policies regarding in teacher support and accountability. Next, we extended prior work using a subsample of the Australian teachers for whom we had matching student data. This second set of results revealed that schools with a greater proportion of low-SES students were more likely to present an Unsupportive school profile. Moreover, the Supportive school profile was associated with higher levels of student-reported instructional support and school-average achievement in reading, mathematics, and science.

## Introduction

Teachers' exposure to a variety of demands and resources at work is known to be associated with important workplace outcomes (e.g., Dicke et al., 2018; Skaalvik and Skaalvik, 2018). Recently, researchers have begun to examine demand-resource profiles to better understand the role played by distinct combinations of work-related demands and resources, and the extent to which these combinations are linked to different outcomes among teachers (e.g., Simbula et al., 2012; Collie and Martin, 2017). Emerging work is also considering these profiles at the school-level. More precisely, researchers have started to examine whether different types of schools can be identified based on the prevalence of different teacher demand-resource profiles (Collie et al., 2020a). The combined identification of teacher and school profiles can be helpful for informing policy and practice to improve teachers' experiences at work, and potentially their students' outcomes as well.

**Figure 1:**
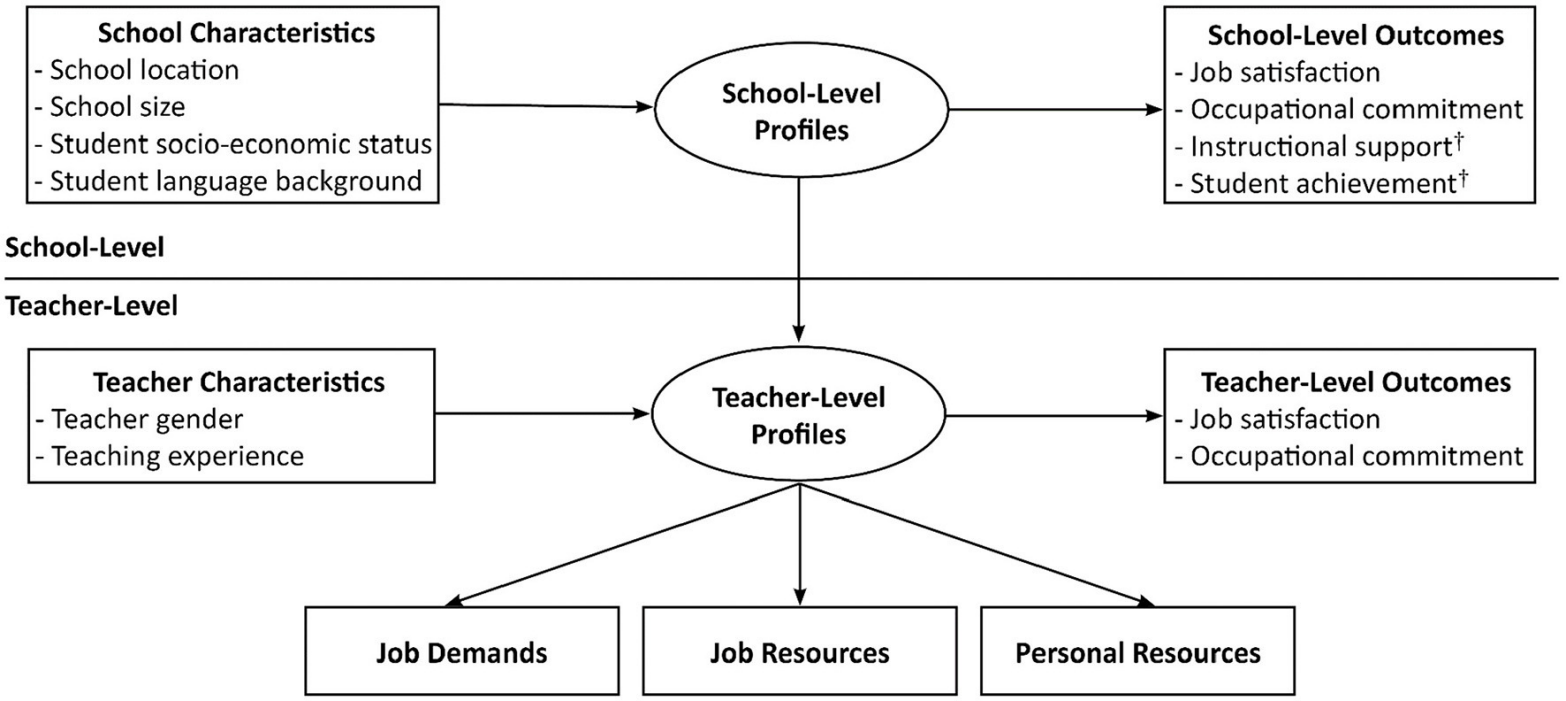
Hypothesized models tested in the study at the teacher- and school-level. In phase one of analysis, teacher-level profiles were identified based on the demands and resources. Then, tests of associations between profile membership and predictors (teacher characteristics) and outcomes (teacher-level outcomes) were conducted. Following this, school-level profiles were identified. Then, tests of associations between profile membership and predictors (school characteristics) and outcomes (school-level outcomes) were conducted. In phase two^†^, analyses involved a subsample of Australian teachers who worked at schools with matched student data, and tests between profile membership and student outcomes (i.e., instructional support, student achievement) were conducted. Not shown here are the tests of profile similarity that were conducted to compare the teacher- and school-level results across countries (see Methods).

Collie et al. (2020a) provided the first ever multilevel person-centered investigation of teacher and school demand-resource profiles, along with an examination of how these profiles were related to teacher- and school-level outcomes. They did so using data collected in the Teaching and Learning International Survey (TALIS) 2013 from Australia and England, which are two countries that have similarities in their educational systems and student populations (for a discussion, see Collie et al., 2020a; see also Fackler et al., 2020). The first goal of the present study is to assess whether Collie et al.'s (2020a) seminal research findings will be replicated among new samples of Australian and English teachers using data collected 5 years later as part of TALIS 2018, and relying on the same measures of job demands (barriers to professional development, disruptive student behavior), job resources (teacher collaboration, input in decision-making), and personal resources (self-efficacy). Given the many changes and increases in complexity that have impacted teachers' work between 2013 and 2018 (discussed in detail below; Guerriero and Révai, 2017), testing for replication using large-scale representative data appears to be critically important for at least two reasons. First, it provides a means to better understand teachers' contemporary experiences. Second, it provides a means to assess whether and how these recent changes may have affected teachers' work orientation. Thus, following from Collie et al. (2020a), the present study seeks to identify teacher and school demand-resource profiles separately in each country, before relying on systematic tests of profile similarity to assess replication across countries. We then consider how these profiles are related to teacher and school characteristics and to outcomes located at both levels of analysis (teacher and school-average job satisfaction and occupational commitment). Figure 1 displays the models under examination.

The second goal of the current study is to extend Collie et al.'s (2020a) research (and other prior work) by examining whether and how the identified school profiles are associated with differences in student-reported outcomes. Examining the extent to which school profiles are linked with students' outcomes has the potential to guide intervention efforts aiming to promote healthy and effective schools—both for teachers and students. In the current study, we examined (school-average) student perceptions of instructional support provided by teachers (autonomy-support, instrumental help, and warmth) and achievement in reading, mathematics, and science. In this second phase, data from a subsample of Australian teachers was used, along with matched student data aggregated at the school-level from the Programme for International Student Assessment (PISA) 2018 study.

### Conceptual Framework and Relevance for Person-Centered Approaches

We rely on the Job Demands-Resources (JD-R) theoretical model (Bakker and Demerouti, 2017) as the conceptual framework for the present study. According to the JD-R framework, all jobs are seen as involving a range of demands and resources that are psychological, social, physical or organizational in nature. Whereas, job demands (e.g., high workload) entail a cost for the employee, and hinder employee functioning at work, job resources (e.g., social support) represent a gain for the employee and help to foster employee positive functioning at work (Bakker and Demerouti, 2017). JD-R theory also establishes that employees bring with them their own personal resources (e.g., self-efficacy), which act as additional types of resources in fostering employee positive functioning at work. Thus, the JD-R theoretical framework assumes that job demands, job resources, and personal resources are all directly associated with important work-related outcomes for the exposed employees, such as motivation, well-being, and performance. In addition to those direct processes, JD-R theory proposes two interactive processes. In the first, which is called the buffering process, job resources are posited to reduce the detrimental impact of job demands on negative outcomes like burnout and stress. In the second, which is called the boosting process and was the focus in the present study, high job demands are posited to enhance the beneficial impact of resources on positive outcomes such as motivation and well-being (Bakker and Demerouti, 2017). The boosting process occurs because job resources become more useful and relevant when demands are high.

A growing body of work has examined teachers' experiences of demands and resources at work. These studies generally show that job demands tend to be associated with poorer work-related outcomes for the exposed teachers (e.g., greater emotional exhaustion, lower well-being, and reduced work commitment; Skaalvik and Skaalvik, 2018), whereas job and personal resources tend to be associated with more positive outcomes (e.g., greater job satisfaction, lower stress; Collie et al., 2018). In addition, research has provided support for the boosting process, whereby job resources appear to share a stronger association with positive outcomes when job demands are high (Bakker et al., 2007). Notably, however, this prior research has largely been conducted using variable-centered approaches (e.g., multiple regression, path analyses) that describe how isolated types of demands and resources are associated with outcomes on average. Because variable-centered approaches rest on the assumption of population-homogeneity, they provide information about associations at a sample-wide level and about particular variables that could be targeted in broad intervention efforts.

More recently, researchers have begun relying on person-centered (e.g., latent profile analysis; Mäkikangas et al., 2018) investigations of the JD-R theoretical framework. Person-centered approaches allow for the identification of different subpopulations (or profiles) of teachers characterized by qualitatively distinct configurations of demands and resources. In this context, person-centered approaches appear to be highly relevant to the development of intervention procedures tailored to distinct profiles of teachers. With respect to JD-R theory, person-centered approaches are particularly relevant to investigating the boosting (and buffering) process (Collie et al., 2020a). Indeed, whereas variable-centered research is able to examine the boosting process *via* tests of interactions among pairs of variables, it is difficult to clearly interpret the results from analyses involving more than three interacting factors. In contrast, person-centered approaches are able to directly consider the combined role played by any number of demands and resources simultaneously. This has practical implications for schools, in which principals typically have to deal with “types” (or profiles) of teachers presenting different characteristics, rather than with the isolated and combined effects of variables. As such, person-centered profiles tend to be readily recognizable in practice. Importantly, person-centered analysis can be used to identify both individual- and school-level profiles, such as teacher and school profiles, making it possible to devise initiatives targeting specific types of teachers, as well as specific types of schools.

### Overview of Prior Research on Demand-Resource Profiles

A small, but growing body of work has identified demand-resource profiles among employees and organizations, with a handful of these studies having more specifically focused on teachers and schools. For example, Simbula et al. (2012) investigated two job resources (professional development and collegial support) and two job demands (role ambiguity and over investment in work) among teachers. Their findings revealed three profiles: high demand-high resource, high demand-low resource, and low demand-high resource. Among an undifferentiated sample of employees, Van den Broeck et al. (2012) identified three similar profiles, along with a fourth (low resource-low demand) in a study focusing on several job demands (e.g., workload, emotional and cognitive demands) and job resources (e.g., social support, autonomy). In terms of organization-level profiles, Mäkikangas et al. (2018) examined department-level demand-resource profiles among university employees. They showed two employee profiles (low demand-high-resource vs. high demand-low resource), along with two department-level profiles: a high stress department (dominated by employees corresponding to the high demand-low resource profile) and a mixed-stress department (characterized a relatively even mix of the two employee profiles). More recently, Collie et al. (2020a) conducted a multi-level investigation of demand-resource profiles among teachers (Level 1) and schools (Level 2). Because this study forms the basis for the current study, we now consider their work in greater detail.

#### Key Findings From an Earlier Study

Collie et al. (2020a) examined five demands and resources that are known to play a major role in the capability of teachers to effectively undertake their work. *Barriers to professional development* is a first type of job demand referring to teachers' exposure to issues preventing them from accessing the training required for their ongoing learning and development (e.g., financial constraints, limited opportunities; OECD, 2009; Broadley, 2010). *Disruptive student behavior* is a second type of job demand referring to teachers' exposure to student off-task behavior that hinders effective instruction (e.g., students refusing to listen, antisocial behaviors; Skaalvik and Skaalvik, 2018). *Teacher collaboration* is a first type of job resource referring to the extent to which teachers are able to efficiently work with their colleagues to plan, develop, teach, and assess student learning (OECD, 2014). *Teacher input in decision-making* is a second type of job resource referring to teachers' perceptions that their school provides them with opportunities to have an input in, and share responsibility for, school decisions (OECD, 2014). Finally, *self-efficacy for teaching* is a personal resource referring to teachers' confidence in their ability to efficiently undertake a range of tasks designed to ensure and maximize student learning (Tschannen-Moran and Woolfolk Hoy, 2001). The present study focuses on the same demands and resources.

Taken together, these five demands and resources reflect relatively common experiences in teaching that impact teachers' ability to be effective and to thrive at work (e.g., Klassen et al., 2012). More precisely, these five factors encompass teachers' interactions with students, colleagues, and school leaders, as well as teachers' own professional development and confidence. Teachers who have positive interactions with others at school, and who feel confident in their abilities, have been found to be more likely to experience positive work-related outcomes (e.g., Klassen et al., 2012). The opposite is true for teachers who have challenging interactions with students or who do not experience agency in relation to their professional growth (e.g., Skaalvik and Skaalvik, 2018).

At the teacher-level (Level 1), Collie et al. (2020a) identified five demand-resource profiles that were equivalent across samples of teachers from Australia and England. The first profile, called the Low-Demand-Flourisher, was characterized by low demands and high resources (both job and personal). The second profile, called the Mixed-Demand-Flourisher, was characterized by low-average demands and high resources. The third profile, the Job-Resourced-Average profile, was characterized by average job demands, above average job resources, and average personal resources. The fourth profile, the Balanced-Average profile reported average levels across all demands and resources. Finally, the Struggler profile reported high demands and low resources. At the school-level (Level 2), Collie et al. (2020a) identified two school profiles: a Supportive school profile comprising relatively equal levels of the Mixed-Demand-Flourisher, Job-Resourced-Average, Struggler, and Low-Demand-Flourisher profiles, and an Unsupportive school profile comprising much higher levels of the Struggler profile. These school profiles were also found to be equivalent in Australia and England.

#### Summary

Taken together, prior research provides growing understanding of the nature of the demand-resource profiles most commonly identified among teachers and schools. At the same time, given the limited number of previous studies, more research is needed to ascertain the extent to which the same number of profiles, characterized by a similar shape, will be identified in new samples, and whether these profiles will display similar outcome implications and associations with predictors. This verification is important in order to formally assess the extent to which previous results can be expected to generalize to new samples of participants, which forms an important pre-requisite to the development of intervention procedures guided by these person-centered results (e.g., Morin et al., 2016). Furthermore, it is also important to consider how these profiles will related to novel predictors and outcomes for extending our understanding of their nomological network.

As noted earlier, the aim of the present study is to replicate Collie et al.'s (2020a) research, but also to extend this earlier study. Replication was undertaken by conducting person-centered analyses on the same set of demands and resources among a similar sample of Australian and English teachers (Level 1) and schools (Level 2) using data collected 5 years after those examined in the Collie et al. (2020a) study. In the upcoming pages, we first outline how changes in policies and practices occurring between 2013 and 2018 might have impacted teachers' work experiences over this time. These changes provide a compelling case for the importance of conducting a replication study. We then more specifically address the various predictors and outcomes considered in the present study, as well as how this study was designed to extend upon Collie et al.'s (2020a) results.

### Recent Changes in Teachers' Work

Although many aspects of teachers' work roles remained similar between 2013 and 2018, there was also (inevitable) ongoing evolution of the teaching profession that resulted in some changes to teachers' experiences of demands and resources at work. Worldwide, there has recently been concern about the growing complexity of the teaching profession and the rising demands being placed upon teachers (e.g., Guerriero and Révai, 2017; OECD, 2019a). In both Australia and England, significant concerns about high workload have been raised by governments (e.g., UK Department for Education, 2018; Parliament of Australia, 2019). Alongside these concerns, there were also policy shifts in Australia and England between 2013 and 2018 that may have impacted teachers' perceptions of work. Thus, both countries implemented new policy priorities toward improving teacher quality, including increases to school funding and the promotion of teachers' professional development and collaboration (OECD, 2019a). This may have led to greater access to professional development and higher levels of collaboration with colleagues. At the same time, (student-reported) disruptive student behavior has recently been found to be higher in Australia and England than in many other OECD countries (OECD, 2019a), and was found to have increased in Australia over the past decade (Thomson et al., 2020). Increases in compliance, accountability, and external evaluation are also apparent in both countries between 2013 and 2018 (OECD, 2019a). These changes might have meant that teachers experienced increases in disruptive behavior and decreases in their ability to provide input in decision-making. Compliance and accountability are also known to be associated with reduced self-efficacy among teachers (von der Embse et al., 2016).

Taken together, although teachers' work roles remained broadly similar from 2013 to 2018, policies and practices changed during this period in a way that might have impacted teachers' experiences, and demand-resource profiles, at work. For example, increases in accountability and compliance (OECD, 2019a) might be accompanied by shifts in teachers' experiences of input and self-efficacy at work, as well as in the way these two types of resources tend to be associated for teachers. Alternatively, if we assume that the identified profiles reflect more enduring differences among teachers, who work in an occupation that is known to experience changes in policies and practices in an ongoing manner, then these changes might not have impacted the nature of the observed demand-resource profiles. Arguably, these two possibilities are both likely. Thus, although we do expect the identified profiles to generalize (which is a critical condition to our ability to use them to guide interventions that will be efficient over time), we leave as an open research question whether this generalizability will be complete, or partial. Furthermore, it is also possible that the changes occurring between 2013 and 2018 might have modified the associations between the observed profiles and the predictors and outcomes. We now turn our attention to these predictors and outcomes.

### Teacher and School Characteristics That Predict Profile Membership

Teacher background characteristics have been shown to predict membership in demand-resource profiles. For example, male teachers and more experienced teachers tend to correspond to less adaptive teacher profiles (e.g., Collie et al., 2020a). Given these results, teacher sex and teaching experience were examined as predictors of the teacher profiles identified in the present study. Little is known, however, in relation to variables involved in the prediction of school profiles. For instance, Mäkikangas et al. (2018) found no associations between departmental size and the department-level profiles identified among university employees. Notably, Collie et al. (2020a) did not examine school-level predictors in their study. In the present study, school-level predictors were thus considered as a way to expand upon Collie et al.'s (2020a) prior work. In light of prior variable-centered research results highlighting the key role played by various school characteristics, this inclusion is deemed to be important. For instance, different school locations are known to come with unique supports and challenges that can impact teachers' workplace experiences (Klassen and Chiu, 2010). For example, schools in less populous locations and with lower socio-economic status typically have less access to teaching resources and professional development opportunities (e.g., Broadley, 2010). Larger schools have been shown to engage in more teacher collaboration (Collie et al., 2020b), but to have lower levels of teacher input (Collie, 2021). Likewise, in English-speaking countries, schools with more students from non-English speaking backgrounds have been shown to have lower levels of teacher collaboration (Collie et al., 2020b). Given these variable-centered associations, it appeared important to consider the extent to which various school characteristics are also associated with school demand-resource profiles.

In sum, the present study first provided an opportunity to verify whether Collie et al.'s (2020a) results regarding the role played by two teacher characteristics (sex, teaching experience) in predicting teachers' membership into the various profiles would generalize in this new sample collected 5 years later. In addition, it also allowed us to expand on these previous results by also considering the role played by four school characteristics (school location, school size, proportion of students from low socio-economic status [SES] backgrounds, and proportion of students from non-English speaking backgrounds) in the prediction of the school-level profiles. We anticipated that the findings obtained at the teacher-level would be similar to those reported by Collie et al. (2020a). At the school-level, based on the previously reported variable-centered results, we hypothesized that schools located in more populous locations, as well as schools with fewer students from low-SES and non-English speaking backgrounds would be more likely to display a Supportive profile. Given mixed findings related to school size, we left the nature of the associations between this school characteristic and the profiles as an open research question.

### Teacher and School Profiles and Associations With Teacher and Student Outcomes

Membership in demand-resource profiles is associated with differences in teachers' work-related outcomes. For example, in Collie et al.'s (2020a) study, the two Flourisher profiles (characterized by average or low demands, and high resources) tended to display the highest levels of job satisfaction (i.e., contentment regarding one's job) and occupational commitment (attachment to one's profession), followed by the two Average profiles (mixed or average resources/demands), and finally by the Struggler profile (high demands, low resources). At the school-level, Collie et al. (2020a) found that the Supportive school profile displayed higher levels on the two outcomes than the Unsupportive school profile. In addition, their results showed that Australian teachers and schools typically reported higher levels on these two outcomes than English teachers and schools. In the current study, we seek to replicate these findings. We also extended Collie et al.'s (2020a) results by considering the school profiles in relation to (school-average) student outcomes (perceptions of instructional support and achievement).

A growing body of research highlights the association between teachers' experiences at work and student outcomes. For example, teachers' job and personal resources have been associated with higher levels of student achievement, motivation, and perceived instructional support (Burić and Kim, 2020). The reverse has been found for job demands (Collie et al., 2020b). To our knowledge, however, prior research has almost universally employed variable-centered approaches to identify links between variables at a sample-wide level. One exception comes from Klusmann et al.'s (2008) research examining personal resource profiles (of engagement and resilience) among teachers. In this research, the authors revealed that more adaptive profiles (high engagement and resilience) tended to be associated with higher levels of perceived instructional support among students than less adaptive profiles.

In the present study, we rely on person-centered analyses to examine the extent to which the identified school demand-resource profiles will be associated with different levels of school-average instructional support as perceived by the students, and with different levels of school-average academic achievement in three areas (reading, mathematics, and science) as assessed in PISA 2018. We consider three types of instructional support: (1) Students' perceptions of autonomy-support, referring to the extent to which teachers are perceived as supporting students' empowerment and self-initiative (Skinner and Belmont, 1993; Ryan and Deci, 2017); (2) Students' perceptions of instrumental help, referring to the extent to which teachers are perceived as using feedback to guide student learning and improvement (Skinner and Belmont, 1993; Ang, 2005); (3) Students' perceptions of teacher warmth, referring to the extent to which teachers are perceived as displaying enjoyment and enthusiasm in relation to teaching and to the subject content (Skinner and Belmont, 1993; Keller et al., 2016). Together, the three types of instructional support encompass key components of well-known models of effective instruction (e.g., Skinner and Belmont, 1993; see also Hamre et al., 2013). Because school characteristics are known to be associated with students' perceptions of instructional support and achievement (e.g., Burns et al., 2019), we also examined the associations between the school profiles and student outcomes while controlling for school characteristics.

Overall, we anticipate that more supportive school profiles would be linked with higher levels on all outcomes. This is because schools where teachers experience more positive relationships and support, and where teachers are more confident in their teaching, are more likely to provide a more supportive and effective learning environment for students (e.g., Burić and Kim, 2020). Likewise, in schools where teachers feel supported at work, students are also more likely to feel supported and to thrive academically (Collie and Martin, 2017).

## Study Overview

The aim of the present study was to replicate and extend prior research by identifying teacher and school demand-resource profiles, along with their predictors and outcomes at the teacher-level (Level 1) and school-level (Level 2). In the first (replication) phase of the study, demand-resource teacher and school profiles were identified among teachers from Australia and then England using multilevel latent profile analyses. Profile similarity tests were then conducted to systematically verify the extent to which the results are comparable across countries. Following this, additional analyses were conducted to assess whether and how the teacher and school profiles are predicted by teacher (sex, teaching experience) or school characteristics (school location, school size, proportion of students from low-SES backgrounds, and proportion of students from non-English speaking backgrounds; with and without controls for predictors), and are associated with teacher or school-average outcomes (job satisfaction and occupational commitment). In the second (extension) phase of the study relying on a subsample of the Australian teachers, along with matched data from students, we ascertained the extent to which the school profiles are predicted by the same school characteristics and are associated with student outcomes (with and without controls for predictors in place). The student outcomes were students' perceptions of instructional support (i.e., autonomy-support, instrumental help, warmth) and achievement in reading, mathematics, and science.

## Methods

### Sample and Procedure

Data used in the present study were from the TALIS 2018 (OECD, 2019c) and PISA 2018 (OECD, 2020) surveys. Institutional review board approval was received for the study and all ethical requirements were complied with in undertaking the study.

#### Phase 1

The Phase 1 sample included 5,439 teachers from 364 schools located in Australia and 2,216 teachers from 149 schools located in England from TALIS 2018. Every 5 years, the Organization for Economic Cooperation and Development (OECD) runs TALIS, which involves comprehensive and nationally-representative data collection among teachers in relation to a range of workplace experiences and perceptions. The TALIS 2018 sample was built using a two-stage probability sampling design to ensure a representative sample of schools and teachers in both countries (for details see OECD, 2019c).

Starting with the Australian sample, participating teachers were 62% female, had an average teaching experience of 15 (*SD* = 11) years, and around half (51%) of them were aged between 30 and 49 years. Most teachers (80%) were working full-time, and almost all (97%) had a bachelor's degree or higher. The Australian teachers taught at ISCED Level 2 (lower secondary) and/or ISCED Level 3 (upper secondary). Over half (61%) of the participating Australian schools were publicly managed, and the majority had fewer than one-third of students from low-SES backgrounds (67%). The majority of schools (57%) had a male principal who had, on average, 8 (*SD* = 7) years of experience as a principal. The schools were located in villages (6%; ≤3,000 people), towns (28%; 3,001–100,000 people), and cities (63%; >100,000 people). There were on average 15 (*SD* = 4) teachers per school.

Participating teachers from England were 65% female, had an average teaching experiences of 13 (*SD* = 12) years, almost two-thirds (62%) of them were aged between 30 and 49 years. Most teachers (80%) were working full-time, and 99% had a bachelor's degree or higher. The entire English sample taught at ISCED Level 2 (lower secondary). Just over one-third (36%) of the participating English schools were publicly managed, and the majority had fewer than one-third of students from low-SES backgrounds (69%). Most schools (59%) had a male principal who had, on average, 6 (*SD* = 5) years of experience as a principal. The schools were located in villages (9%; ≤3,000 people), towns (54%; 3,001–100,000 people), and cities (38%; >100,000 people). There were on average 15 (*SD* = 4) teachers per school.

#### Phase 2

A subsample of 2,099 Australian teachers from 130 schools was examined, along with matched data from 2,048 students who participated in PISA 2018. Using the OECD (2019c) TALIS-PISA link, it was possible to match the teacher and student data at the school level. The TALIS-PISA linked data were only available for the ISCED Level 3 (upper secondary) teachers in the Australian teacher sample (England did not participate in the TALIS-PISA link). Teachers from this subsample were 60% female, had an average teaching experience of 15 (*SD* = 11) years, and 51% of them were aged between 30 and 49 years. Most schools (59%) had a male principal who had, on average, 8 (*SD* = 7) years of experience as a principal. The schools were located in villages (8%; ≤3,000 people), towns (28%; 3,001–100,000 people), and cities (64%; >100,000 people). The students were 51% female with an average age of 15.80 years (*SD* = 0.30) years. In the subsample, there were on average 16 (*SD* = 4) teachers and 18 (*SD* = 5) students per school.

### Measures

Teacher and school measures were drawn from the TALIS 2018 Teacher and Principal Questionnaires, respectively (OECD, 2019c). Student measures were drawn from the PISA 2018 Student Questionnaire (OECD, 2020; see Supplementary Material for items). The profile indicator variables and the teacher characteristics were modeled at the teacher-level. The teacher outcomes (job satisfaction and occupational commitment) were modeled at the teacher- and school-level. School characteristics and student outcomes were modeled only at the school-level.

#### Job Demands

*Barriers to professional development* was assessed with 6 items from the TALIS 2018 “Barriers to Professional Development” scale (e.g., “Professional development is too expensive/unaffordable”). *Disruptive student behavior* was assessed with items from the TALIS 2018 “Your Teaching” scale (3 items; e.g., “I lose quite a lot of time because of students interrupting the lesson”). For both scales, items were scored from 1 (Strongly disagree) to 4 (Strongly agree). Reliability was assessed with coefficient omega^1^ and was adequate across both countries for barriers to professional development (ω = 0.755) and disruptive student behavior (ω = 0.908). The barriers to professional learning scale displayed 7% variance at the school-level (intraclass correlation [ICC] = 0.074). Although this is somewhat modest, this proportion is sufficient to support the need for multilevel analyses (Bliese et al., 2018). The disruptive student behavior scale also demonstrated adequate variance at the school-level (ICC = 0.169).

#### Job Resources

*Teacher collaboration* was assessed with items from the TALIS 2018 “Teaching in General” scale (3 items; e.g., “On average, how often do you do the following in this school? Exchange teaching materials with colleagues”). Items were scored on a scale from 1 (Never) to 6 (Once a week or more). Reliability estimates were adequate across both countries (ω = 0.719) and there was adequate variance at the school-level (ICC = 0.195).

*Teacher input in decision-making* was assessed with items from the TALIS 2018 “School Climate” scale (3 items; e.g., “This school provides staff with opportunities to actively participate in school decisions”). Items were scored from 1 (Strongly disagree) to 4 (Strongly agree). Reliability was satisfactory (ω = 0.838) and there was adequate variance at the school-level (ICC = 0.156).

#### Personal Resources

*Teacher self-efficacy* was assessed with items from the TALIS 2018 “Teaching in General” scale, which encompasses three types of self-efficacy: self-efficacy for classroom management (4 items; e.g., “Control disruptive behavior in the classroom”), self-efficacy for instruction (4 items; e.g., “Vary instructional strategies in my classroom”), and self-efficacy for student engagement (4 items; e.g., “Motivate students who show low interest in school work”). Items all followed the stem “In your teaching, to what extent can you do the following?” and were scored from 1 (Not at all) to 4 (A lot). Reliability for the three factors of self-efficacy was satisfactory (ω = 0.775–855). For reasons of parsimony and because the self-efficacy factors were quite highly intercorrelated (*r*'s = 0.60–0.63), self-efficacy was modeled as a single higher-order factor (ω = 0.824) displaying modest, but sufficient variance at the school-level (ICC = 0.066; Bliese et al., 2018).

#### Teacher Characteristics

*Teacher sex* was coded 0 for female, 1 for male. *Teaching experience* was a continuous variable measured in years.

#### School Characteristics

School location was coded 1 for a village (≤3,000 people), 2 for a town (3,001–100,000 people), or 3 for a city (>100,000 people). School size was coded as 1 (under 250 students), 2 (250–400 students), 3 (500–749 students), 4 (750–999 students), or 5 (>1,000 students). Proportion of students from low socio-economic status (SES) backgrounds and proportion of students from non-English speaking backgrounds (NESB) were coded as 1 (0%), 2 (1–10%), 3 (11–30%), 4 (31–60%), or 5 (more than 60%).

#### Teacher Outcomes

*Job satisfaction* (3 items; e.g., “All in all, I am satisfied with my job”) and *occupational commitment* (4 items; e.g., “If I could decide again, I would still choose to work as a teacher”) were assessed with items from the TALIS 2018 “About Your Job” scale. For both outcomes, items were scored from 1 (Strongly disagree) to 4 (Strongly agree). Both outcomes were modeled at the teacher- and school-levels. Reliability was satisfactory at the teacher-level (ω = 0.847 for job satisfaction; ω = 0.831 for occupational commitment) and the school-level (ω = 0.989 for job satisfaction; ω = 0.950 for occupational commitment). Job satisfaction (ICC = 0.138) and occupational commitment (ICC = 0.047) both had enough variability at the school-level (Bliese et al., 2018).

#### Student Outcomes

Instructional support was assessed with three scales reported by students and aggregated at the school-level in analyses. *Autonomy-support* was assessed with the PISA 2018 “Teacher Support” scale (3 items; e.g., “The teacher listened to my view on how to do things”). Items were scored from 1 (Strongly disagree) to 4 (Strongly agree). Reliability was satisfactory at the student- (ω_Student_ = 0.904) and school-level (ω_School_ = 0.990), and school-level variability was sufficient (ICC = 0.036).

*Instrumental help* was assessed with items from the PISA 2018 “Perceived Feedback” scale (3 items; e.g., “The teacher gives me feedback on my strengths in this subject”). Items were scored from 1 (Never or almost never) to 4 (Every lesson or almost every lesson). Reliability was satisfactory at the student-level (ω_Student_ = 0.913) and school-level (ω_School_ = 0.979), and school-level variability was sufficient (ICC = 0.035).

*Teacher warmth* was assessed with the 4 items in the PISA 2018 “Perceived Teacher Interest” scale (4 items; e.g., “It was clear to me that the teacher liked teaching us”). Items were scored from 1 (Strongly disagree) to 4 (Strongly agree). Reliability was satisfactory at the student-level (ω_Student_ = 0.887) and school-level (ω_School_ = 0.984), and school-level variability was sufficient (ICC = 0.078).

*School-average achievement* in the areas of reading, mathematics, and science was measured *via* the Bayesian plausible values provided for each student in PISA 2018 and aggregated at the school-level in analyses. In PISA 2018, the reading test assessed students' capacity in locating information, comprehension and integrating knowledge, and evaluating and reflecting (OECD, 2019b). The mathematics test covered three areas: recognizing and identifying appropriate mathematical approaches; employing mathematical concepts and facts; and, interpreting, applying, and evaluating mathematical outcomes (for full details, see OECD, 2019b). The science test assessed three domains: understanding of different personal, local, national, and global contents; content, procedural, and epistemic knowledge relating to science facts, concepts, and theories; and, competencies including explaining, evaluating, and interpreting science tasks and data (OECD, 2019b). For each student and in each of the achievement areas, PISA produces 10 plausible values (for further details, see OECD, 2020). To accurately employ these scores, models need to be estimated 10 times (each with a different set of plausible values; OECD, 2020). Results are then aggregated using the Rubin (1987) strategy to obtain unbiased parameter estimates and standard errors. To calculate reliability, the 10 plausible values for each subject were used as indicators of a latent factor. Estimates were satisfactory at the student-level and school-level for reading achievement (ω_Student_ = 0.991, ω_Sschool_ = 0.999), mathematics achievement (ω_Student_ = 0.976, ω_Sschool_ = 0.997), and for science achievement (ω_Student_ = 0.984, ω_Sschool_ = 0.998). Reading achievement (ICC = 0.199), mathematics achievement (ICC = 0.245), and science achievement (ICC = 0.208) demonstrated adequate variance at the school-level.

### Data Analysis

All analyses were conducted using M*plus* 8.4 (Muthén Muthén, 2019). Teacher (TCHWGT), student (W_FSTUWT), and school weights (SCHWGT) were applied to account for the probabilities of selection and participation at the different stages of sampling (see OECD, 2019b,c for details). The clustering of teachers within schools was accounted for in single-level modeling by using the M*plus* design-based correction procedures (Asparouhov, 2005). The robust maximum likelihood (MLR) estimator was used in all models. This estimator is robust to non-normality and to complex data structures. The limited amount of missing data was handled using full information maximum likelihood (FIML) estimation procedures (Enders, 2010). More precisely, missing data for teacher-level variables (demands, resources, teacher outcome, and teacher characteristics) were 1–5% (except disruptive student behavior, which was 16%). Missing data for school characteristics were 3–6%. Missing data for aggregated student-related outcomes were 7% for instructional support and 0% for achievement.

### Preliminary Analyses

Preliminary confirmatory factor analyses (CFA) were conducted to ascertain the psychometric properties of our measures, and their measurement invariance (Millsap, 2011) across countries. Separate sets of models were estimated for the profile indicators (the five demands and resources), the teacher outcomes, and the students' instructional support outcomes. Because the profile indicator variables are only estimated at the teacher level, and then the profiles are used to estimate school-level profiles, the measurement models underpinning these indicators were estimated at the teacher level. In contrast, because the teacher outcomes were modeled at both the teacher and school levels, these preliminary analyses relied on multilevel-CFA. Finally, because student outcomes were reported by the students, but used at the school level (student and teachers were only matched at the school level in the TALIS-PISA link), these preliminary analyses relied on multilevel-CFA conducted at the student and school levels. The estimates of composite reliability (omega) and intraclass correlation coefficients (ICC) reported in the “Measures” sections were calculated from the most invariant of these measurement models (factor loadings, intercepts, residuals, covariances, variances, and means). Factor scores were saved from the most invariant of these measurement models and used as input for our main analyses, together with manifest scores reflecting the teacher characteristics, school characteristics, and aggregated achievement outcomes. Additional details on these preliminary analyses, which supported the complete invariance of our measures and the isomorphism (equality) of our factor loadings across levels for all multilevel analyses are reported in the first section of the Supplementary Material. Table 1 shows the reliability coefficients and descriptive statistics at the teacher-level and school-level. Latent correlations among the variables from the most invariant models are available in Supplementary Table 2. Prior to undertaking our main analyses, a multigroup (across countries) baseline model using the factor scores of the profile indicators was estimated to standardize the sampling weights separately for each country using procedures outlined in Collie et al. (2020a). These standardized weights were then used in all analyses outlined below.

**Table 1:** Reliabilities and descriptive statistics for both countries.

		**Australia**		**England**	
	**ω**	***M***	***SD***	***M***	***SD***
**Teacher-level**					
Barriers to professional development	0.755	2.247	0.579	2.349	0.583
Disruptive student behavior	0.908	2.092	0.775	2.011	0.776
Teacher collaboration	0.719	4.962	0.971	4.662	1.063
Teacher input	0.838	2.755	0.620	2.719	0.595
Teacher self-efficacy	0.824	3.246	0.466	3.340	0.451
Job satisfaction	0.847	3.157	0.589	3.022	0.612
Occ. commitment	0.831	3.122	0.628	2.841	0.691
**School-level**					
Job satisfaction	0.989	3.153	0.253	3.020	0.288
Occ. commitment	0.950	3.126	0.210	2.844	0.230
Autonomy-support	0.990	2.865	0.239	—	—
Instrumental help	0.979	2.600	0.263	—	—
Teacher Warmth	0.984	1.786	0.286	—	—
Reading achievement	0.999	497	53	—	—
Mathematics achievement	0.997	486	44	—	—
Science achievement	0.998	498	47	—	—

*For all teacher-related factors, coefficient omega (ω) is reported for both countries as it was taken from the most invariant measurement invariance test. For the student outcomes, omega involves only the Australian sample for the TALIS-PISA link (given England did not participate in the TALIS-PISA link; however, see Sizmur et al., 2019 for details about school-level outcomes for England). The achievement values are reported as whole numbers as per OECD guidelines (OECD, 2019b). Occ. commitment, Occupational commitment*.

### Phase 1: Single-Level and Multilevel LPA

This initial phase of the study seeks to replicate Collie et al.'s (2020a) study using analytical procedures extensively documented in that previous study. For this reason, we only provide a brief summary of the analytic steps here, and refer interested readers to the Supplementary Material of the Collie et al. (2020a) study for additional details. First, single-level latent profile analyses (LPA) were used to estimate teacher profiles based on the means and variance of the profile indicators at Level 1 (L1). Once the optimal representation of teachers' profile was identified, multilevel-LPA were used to identify school profiles based on the relative frequency of occurrence of these teacher profiles at the school level. For both single-level and multilevel LPA, we estimated solutions including 1 to 8 profiles, separately for the two countries. Each model was estimated using 10,000 random sets of start values, 1,000 iterations, and 100 final stage optimizations. We verified that the best log-likelihood value was properly replicated for all models.

Several indices were employed to assess the relative adequacy of the models, along with elbow plots reflecting the decrease in the value of these indicators as a function of added profiles (e.g., Morin and Litalien, 2019). More precisely, we relied on the Akaike information criterion (AIC) and its consistent version (CAIC), and on the Bayesian information criterion (BIC) and its sample size-adjusted version (SSA-BIC). For these indices, a lower value indicates better fit. Because these values are sample size dependent and thus often fail to converge on a specific solution, we consider a graphical display of these indicators (i.e., an elbow plot). The point at which the drop in the value of these indicators noticeably flattens can be used to guide model selection (Morin et al., 2016). For the single-level models, we also report the *p*-value associated with the adjusted Lo–Mendel–Rubin Likelihood Ratio Test (*p*LMR; this indicator is not available for multilevel-LPA). A statistically significant value on this test supports the value of a solution in relation to a solution with one fewer profile. Alongside these indices, we used parsimony, conceptual relevance, and statistical adequacy to help determine the optimal solution.

After determining the optimal solution separately for each country, at both levels, we undertook tests of profile similarity to ascertain the extent to which the profile solutions could be considered to be comparable across the two countries (Morin et al., 2016). These tests were first conducted for the single-level LPA (Morin et al., 2016) and then for the multilevel LPA starting from the most similar single-level LPA solution (Collie et al., 2020a). More precisely, at the teacher level, we estimated models of configural (i.e., same numbers of profiles), structural (i.e., same within-profile means on the profile indicators), dispersion (i.e., same within-profile means and variances on the profile indicators), and distributional (i.e., same within-profile means and variances, and same profile sizes) similarity across countries (Morin et al., 2016). At the school level, we estimated models of configural, structural, and distributional similarity (Collie et al., 2020a), as no variance components are involved in the estimation of L2 profiles defined on the basis of the relative frequency of occurrence of L1 profiles.

Three additional tests were then estimated to examine the equivalence of the associations between: (a) the predictors (i.e., teacher characteristics at L1: sex and teaching experience; school characteristics at L2: school location, school size, proportion of low-SES students, and proportion of NESB students) and the likelihood of profile membership (predictive similarity; Morin et al., 2016); (b) the profiles and the outcomes (i.e., L1 and L2 job satisfaction and occupational commitment; explanatory similarity; Morin et al., 2016); and (c) the profiles and the outcomes while controlling for the effects of the predictors (adjusted-explanatory similarity; derived from the profile-based ANCOVA approach of McLarnon and O'Neill, 2018). The L2 predictive similarity tests and the L1 and L2 adjusted-explanatory similarity tests represent extensions to the Collie et al. (2020a) study. We provide annotated input syntax for these tests in the Supplementary Material.

For these tests, profile similarity is supported as long as two out of the four aforementioned information criteria are lower for a solution when compared to the previous one from the sequence of profile similarity tests. Predictors and outcomes were directly incorporated into the retained (most similar) solution from the previous steps at L1 as per Morin et al. (2016), and at L2 using the manual three-step approach developed by Collie et al. (2020a) from early work conducted by Litalien et al. (2019) and Morin and Litalien (2017). This approach at L2 was necessary given the way the multilevel LPA was conducted (where the L1 profiles are “predicted” by the L2 profiles; see Collie et al., 2020a). Associations with predictors were assessed using a multinomial logistic link function (i.e., the impact of predictors on the likelihood of membership into each profile relative to all other profiles was estimated; Vermunt, 2010), whereas associations with outcomes were assessed using mean comparisons implemented with the multivariate delta method (Raykov and Marcoulides, 2004). Associations with outcomes while controlling for predictors used the same methods, but also involved regressing the outcomes on the background characteristics (McLarnon and O'Neill, 2018). For interested readers, annotated input files are provided in the Supplementary Material of Collie et al.'s (2020a) article, except for tests of L2 predictive similarity and L1 and L2 adjusted-explanatory similarity, which are provided in our Supplementary Material.

### Phase 2: School-Level Profiles and Student Outcomes in the Australian Subsample

Phase 2 involved an extension of the Collie et al. (2020a) study to consider how the school profiles identified in Phase 1 were associated with predictors and student outcomes among a subsample of the Australian teachers. The baseline model used in these analyses was specified (i.e., constrained) to be identical to the most similar model retained in Phase 1, using the manual three-step procedures outlined in Collie et al. (2020a). Then, three separate analyses were conducted that paralleled Phase 1. For the first analysis, we examined the extent to which the four school characteristics (i.e., school location, school size, proportion of students from low-SES backgrounds, and proportion of students from NESB backgrounds) predicted the likelihood of profile membership (Vermunt, 2010).

For the second analysis, we examined the extent to which the school-level profiles are associated with different levels of the student-reported outcomes using mean comparisons implemented with the multivariate delta method (Raykov and Marcoulides, 2004). The model was run 10 times to account for the 10 Bayesian plausible values provided in PISA for each of the achievement outcomes. The estimates and *p*-values were pooled using the Rubin (1987) strategy (which is automated in the M*plus* 8.4 statistical package; Muthén Muthén, 2019) to obtain unbiased parameter estimates. The third analysis examined associations between profile membership and the student outcomes while controlling for the school characteristics using the previously described approach (McLarnon and O'Neill, 2018).

## Results

### Phase 1: Single-Level LPA

#### Profile Identification and Description

**Figure 2:**
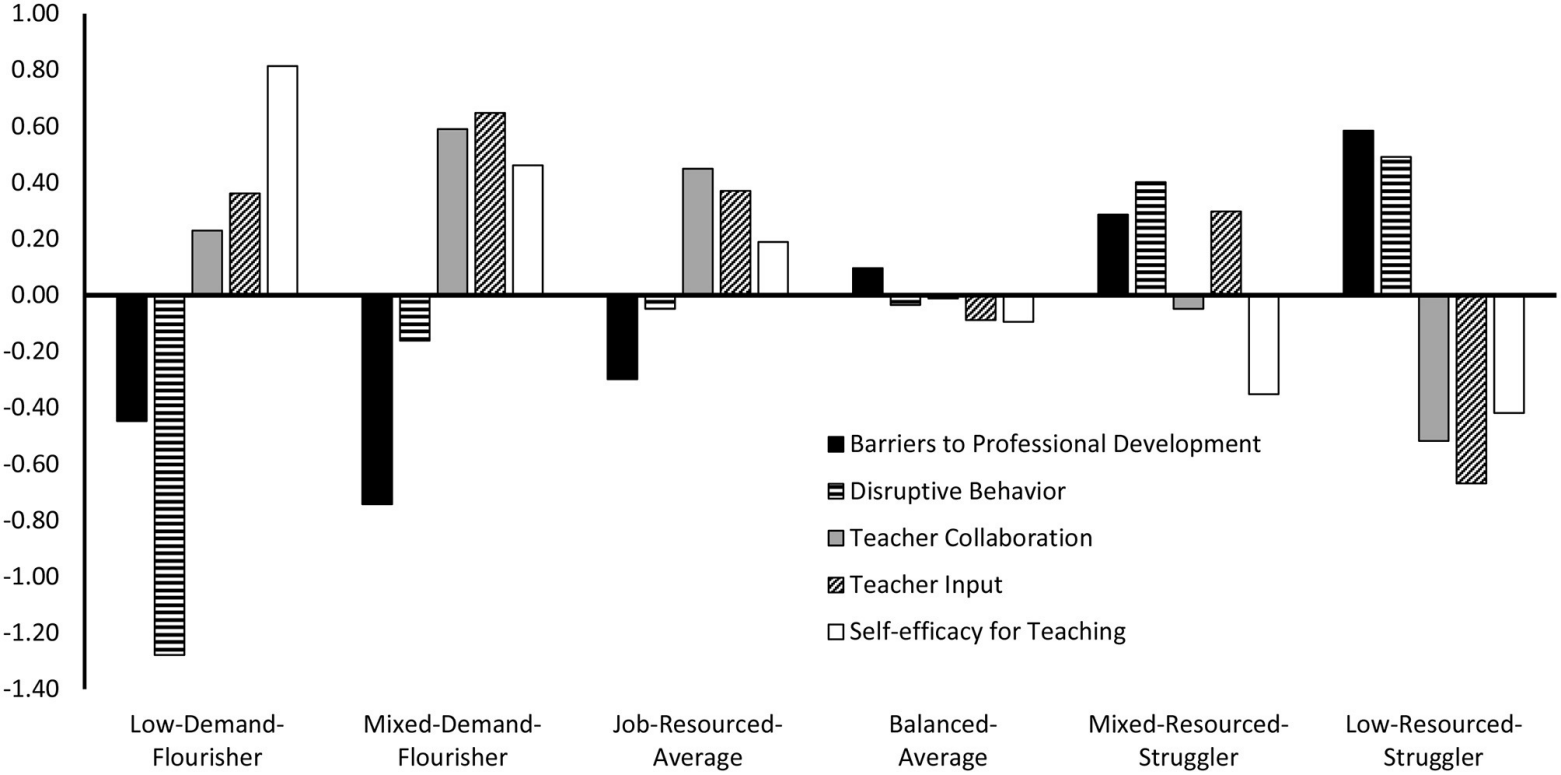
Single-level LPA results of distributional similarity showing teacher profiles for both countries.

Model fit statistics for the solutions involving 1 through 8 profiles estimated separately in Australia and England are reported in Table 2, and corresponding elbow plots are reported in Supplementary Figure 1. For both countries, all information criteria kept on decreasing with the addition of profiles to the solution. The *p*LMR suggested a 6 profile solution in Australia, and either a 4, or 6 profile solution in England. Examination of the elbow plots revealed a slight flattening around 5-and 6 profiles in both countries. In summary, these statistics generally suggest that the optimal solution should include somewhere between 5 and 6 profiles. These two possible solutions, together with the adjacent 4 and 7 profile solutions, were thus further examined for conceptual relevance, parsimony, and meaningfulness. Examination of the 6 profile solution revealed a high level of similarity across country, thus providing preliminary support for configural similarity. When we compared the 5 profile solution to the 6 profile solution, it was clear that the additional profile was meaningful in both countries and presented a differentiated shape compared with the other profiles. In contrast, the 4 profile solution lacked the nuance that was evident in the other solutions, whereas the 7 profile solution did not add anything new (simply resulting in the arbitrary division of one profile into two very similar ones). The 6 profile solution was thus retained for both countries, and submitted to more systematic tests of profile similarity. The results from the L1 tests of profile similarity conducted across the two countries are reported in Table 3, and support the complete similarity (configural, structural, dispersion, and distributional) of the solution across Australia and England. A graphical representation of this final 6 profile solution of distributional similarity is presented in Figure 2, and detailed results associated with this solution are reported in Supplementary Table 6.

**Table 2:** Fit statistics and entropy for Australia and England.

	**Log-likelihood**	**Free Parameters**	**AIC**	**CAIC**	**BIC**	**SSA-BIC**	***p*LMR**	**Entropy**
**Australia—Single-level**								
1 profile	−35132.680	10	70285.361	70361.374	70351.374	70319.597	—	—
2 profiles	−33104.629	21	66251.259	66410.887	66389.887	66323.156	<0.01	0.845
3 profiles	−31391.072	32	62846.143	63089.387	63057.387	62955.701	<0.01	0.771
4 profiles	−30259.995	43	60605.990	60932.848	60889.848	60753.208	<0.01	0.810
5 profiles	−29249.431	54	58606.862	59017.335	58963.335	58791.740	<0.01	0.823
6 profiles	−28773.371	65	57676.743	58170.830	58105.830	57899.281	<0.01	0.832
7 profiles	−28388.420	76	56928.841	57506.543	57430.543	57189.039	<0.01	0.843
8 profiles	−28121.379	87	56416.757	57078.075	56991.075	56714.616	*ns*	0.826
**Australia—Multilevel**								
1 profile	−9251.758	5	18513.517	18551.523	18546.523	18530.635	—	0.709
2 profiles	−9174.344	11	18370.688	18454.303	18443.303	18408.349	—	0.681
3 profiles	−9127.009	17	18288.018	18417.241	18400.241	18346.221	—	0.670
4 profiles	−9113.675	23	18273.349	18448.180	18425.180	18352.093	—	0.696
5 profiles	−9102.291	29	18262.580	18483.019	18454.019	18361.867	—	0.676
6 profiles	−9093.576	35	18257.152	18523.199	18488.199	18376.980	—	0.692
7 profiles	−9085.710	41	18253.421	18565.076	18524.076	18393.791	—	0.693
8 profiles	−9078.078	47	18250.155	18607.419	18560.419	18411.068	—	0.702
**England—Single-level**								
1 profile	−14418.504	10	28857.008	28924.042	28914.042	28882.271	—	—
2 profiles	−13573.799	21	27189.598	27330.371	27309.371	27242.651	<0.01	0.838
3 profiles	−12846.487	32	25756.973	25971.484	25939.484	25837.815	<0.01	0.890
4 profiles	−12330.889	43	24747.779	25036.028	24993.028	24856.410	<0.01	0.809
5 profiles	−11988.036	54	24084.071	24446.058	24392.058	24220.492	*ns*	0.821
6 profiles	−11801.073	65	23732.147	24167.871	24102.871	23896.357	*ns*	0.825
7 profiles	−11660.384	76	23472.767	23982.230	23906.230	23664.767	<0.01	0.819
8 profiles	−11484.460	87	23142.920	23726.121	23639.121	23362.709	*ns*	0.830
**England—Multilevel**								
1 profile	−3712.827	5	7435.654	7469.171	7464.171	7448.286	—	0.720
2 profiles	−3682.214	11	7386.428	7460.166	7449.166	7414.217	—	0.697
3 profiles	−3673.464	17	7380.929	7494.887	7477.887	7423.876	—	0.667
4 profiles	−3668.075	23	7382.150	7536.330	7513.330	7440.255	—	0.676
5 profiles	−3663.898	29	7385.796	7580.197	7551.197	7459.059	—	0.681
6 profiles	−3660.468	35	7390.935	7625.556	7590.556	7479.356	—	0.696
7 profiles	−3658.564	41	7399.127	7673.969	7632.969	7502.705	—	0.707
8 profiles	−3657.222	47	7408.445	7723.507	7676.507	7527.181	—	0.693

*AIC, Akaike Information Criteria; CAIC, Consistent Akaike Information Criteria; BIC, Bayesian Information Criteria; SSA-BIC, sample-size-adjusted Bayesian Information Criteria; pLMR, p-value of the Lo–Mendell–Rubin Likelihood Ratio Test; ns, non-significant*.

**Table 3:** Tests of profile similarity across Australia and England.

	**Log-Likelihood**	**Free Parameters**	**AIC**	**CAIC**	**BIC**	**SSA-BIC**	**Entropy**
**Single-level LPA**							
Configural	−45218.140	131	90698.281	91738.828	91607.829	91191.538	0.876
Structural (means)	−45336.164	101	90874.328	91676.583	91575.583	91254.626	0.878
Dispersion (means and variances)	−45367.694	71	90877.387	91441.349	91370.348	91144.725	0.878
Distributional (means, variances, size)	−45388.761	66	90909.523	91433.768	91367.768	91158.034	0.877
**Predictive similarity**							
Unconstrained across country	−45027.831	26	90107.662	90314.054	90288.054	90205.431	0.878
Constrained across country	−45047.773	16	90127.546	90254.556	90238.556	90187.711	0.878
**Explanatory similarity**							
Unconstrained across country	−53597.303	32	107258.606	107512.786	107480.785	107379.096	0.888
Constrained across country	−53635.126	20	107310.253	107469.114	107449.115	107385.559	0.888
**Adjusted-explanatory similarity**							
Unconstrained across country	−50513.698	47	101121.395	101494.488	101447.488	101298.132	0.885
Constrained across country	−50553.427	35	101176.854	101454.689	101419.689	101308.466	0.885
**Multilevel LPA**							
Configural	−13172.195	23	26390.390	26573.082	26550.082	26476.993	0.754
Structural (proportion of L1 profiles)	−13191.688	13	26409.376	26512.636	26499.637	26458.325	0.745
Distributional (proportion of L2 profiles)	−13194.335	12	26412.670	26507.987	26495.987	26457.854	0.744
**Predictive similarity**							
Unconstrained across country	−16218.676	18	32473.353	32616.328	32598.329	32541.129	0.767
Constrained across country	−16236.084	14	32500.169	32611.372	32597.373	32552.883	0.757
**Explanatory similarity**							
Unconstrained across country	−12434.810	12	24893.621	24988.937	24976.938	24938.805	0.792
Constrained across country	−12545.187	8	25106.375	25169.919	25161.919	25136.497	0.785
**Adjusted-explanatory similarity**							
Unconstrained across country	−15263.750	33	30593.501	30717.756	30822.624	30855.623	0.771
Constrained across country	−15366.656	29	30791.313	30900.507	30992.663	31021.662	0.773

*AIC, Akaike Information Criteria; CAIC, Consistent Akaike Information Criteria; BIC, Bayesian Information Criteria; SSA-BIC, sample-size-adjusted Bayesian Information Criteria*.

Teachers corresponding to Profile 1 (11% of the sample) reported low barriers to professional development, very low disruptive behavior, above average teacher collaboration and teacher input, and high self-efficacy. This profile was thus labeled *Low-Demand*-*Flourisher* to reflect this adaptive blend of low job demands and high job and personal resources. Teachers corresponding to Profile 2 (17% of the sample) reported very low barriers to professional development, below average disruptive behavior, and high teacher collaboration, teacher input, and self-efficacy. This profile was labeled *Mixed-Demand-Flourisher* because of the consistently high levels of the job and personal resources.

Teachers corresponding to Profile 3 (11% of the sample) reported low barriers to professional development, slightly below average disruptive behavior, high teacher collaboration, high teacher input, and above average self-efficacy. This profile was thus labeled *Job-Resourced-Average* to reflect the low to average job demands, coupled with average self-efficacy, but high job resources. Teachers corresponding to Profile 4 (14% of the sample) reported slightly above average barriers to professional development, slightly below average disruptive behavior, average teacher collaboration, and slightly below average teacher input and self-efficacy. We labeled this profile *Balanced-Average* to reflect the matching average levels observed across all demands and resources.

Teachers corresponding to Profile 5 (11% of the sample) reported high barriers to professional development and disruptive behavior, slightly below average teacher collaboration, high teacher input, and low self-efficacy. We labeled this profile *Mixed-Resourced-Struggler* to reflect the mixed levels of resources and high demands. Teachers corresponding to Profile 6 (36% of the sample) reported high barriers to professional development and disruptive behavior, and low teacher collaboration, teacher input, and self-efficacy. We labeled this profile *Low-Resourced-Struggler* to reflect this blend of high job demands, and low job and personal resources.

#### Profile Prediction and Outcomes

The results from the tests of predictive (associations with predictors), explanatory (associations with outcomes), and adjusted-explanatory (associations with outcomes controlling for predictors) similarity (Table 3) supported the equivalence of the associations between the profiles, their predictors, and their outcomes both without and with controls for the predictors in place. The results from these analyses are reported in Table 4 for the predictors, and in Table 5 for the outcomes and the outcomes adjusted for the predictors.

**Table 4:** The role of teacher characteristics in predicting profile membership in both countries (Single-level LPA).

	***b***	***SE***	***OR***	***b***	***SE***	***OR***
	**Low-Demand-Flourisher vs. Mixed-Demand-Flourisher**			**Low-Demand-Flourisher vs. Job-Resourced-Average**		
Sex (F/M)	−0.042	0.107	0.959	−0.073	0.121	0.929
Teaching experience	0.039^**^	0.005	1.040	0.039^**^	0.005	1.039
	**Low-Demand-Flourisher vs. Balanced-Average**			**Low-Demand-Flourisher vs. Mixed-Resourced-Struggler**		
Sex (F/M)	−0.442^**^	0.107	0.643	−0.237	0.121	0.789
Teaching experience	0.038^**^	0.005	1.039	0.057^**^	0.005	1.058
	**Low-Demand-Flourisher vs. Low-Resourced-Struggler**			**Mixed-Demand-Flourisher vs. Job-Resourced-Average**		
Sex (F/M)	−0.314^**^	0.197	0.730	−0.032	0.116	0.979
Teaching experience	0.049^**^	0.004	1.050	−0.001	0.005	0.999
	**Mixed-Demand-Flourisher vs. Balanced-Average**			**Mixed-Demand-Flourisher vs. Mixed-Resourced-Struggler**		
Sex (F/M)	−0.400^**^	0.102	0.670	−0.195	0.112	0.823
Teaching experience	−0.001	0.005	0.999	0.017^**^	0.005	1.017
	**Mixed-Demand-Flourisher vs. Low-Resourced-Struggler**			**Job-Resourced-Average vs. Balanced-Average**		
Sex (F/M)	−0.273^**^	0.087	0.761	−0.369^**^	0.111	0.692
Teaching experience	0.009^*^	0.004	1.009	0.001	0.006	1.000
	**Job-Resourced-Average vs. Mixed-Resourced-Struggler**			**Job-Resourced-Average vs. Low-Resourced-Struggler**		
Sex (F/M)	−0.163	0.128	0.849	−0.241^*^	0.095	0.786
Teaching experience	0.018^**^	0.006	1.018	0.010^*^	0.004	1.010
	**Balanced-Average vs. Mixed-Resourced-Struggler**			**Balanced-Average vs. Low-Resourced-Struggler**		
Sex (F/M)	0.205	0.119	1.228	0.128	0.088	1.136
Teaching experience	0.018^**^	0.006	1.018	0.010^*^	0.004	1.010
	**Mixed-Resourced-Struggler vs. Low-Resourced-Struggler**					
Sex (F/M)	−0.078	0.104	0.925			
Teaching experience	−0.008	0.005	0.992			

* *p ≤ 0.05*;

** *p ≤ 0.01*;

*b, multinomial logistic regression coefficient; SE, standard error of the coefficient; OR, odds ratio. For sex, females were coded 0 and males were coded 1*.

**Table 5:** Means of teacher-level outcomes from explanatory similarity test and adjusted-explanatory similarity test (Single-level LPA).

	**Low-Demand—Flourisher *M*** **(95% CI)**	**Mixed-Demand—Flourisher *M*** **(95% CI)**	**Job-Resourced—Average *M*** **(95% CI)**	**Balanced—Average *M*** **(95% CI)**	**Mixed-Resourced—Struggler *M*** **(95% CI)**	**Low-Resourced—Struggler *M*** **(95% CI)**
**Explanatory similarity test**						
Job satisfaction	0.217 (0.181, 0.253)	0.321 (0.296, 0.347)	0.096 (0.062, 0.129)	−0.040^a^ (−0.071, −0.008)	−0.052^a^ (−0.087, −0.017)	−0.263 (−0.283, −0.243)
Occupational commitment	0.250 (0.216, 0.284)	0.358 (0.334, 0.381)	0.122 (0.091, 0.154)	−0.023^a^ (−0.052, 0.006)	0.003^a^ (−0.027, 0.032)	−0.307 (−0.325, −0.288)
**Adjusted-explanatory similarity test (with controls for predictors)**						
Job satisfaction	0.267 (0.219, 0.315)	0.308 (0.265, 0.351)	0.159 (0.117, 0.202)	0.019^a^ (−0.024, 0.062)	0.003^a^ (−0.040, 0.046)	−0.146 (−0.184, −0.107)
Occupational commitment	0.246^b^ (0.200, 0.292)	0.306^b^ (0.268, 0.345)	0.127 (0.087, 0.166)	−0.026^a^ (−0.067, 0.015)	0.005^a^ (−0.036, 0.045)	−0.269 (−0.303, −0.236)

*Superscript values indicate a mean comparison that was not significantly different across the profiles with the same superscript value across a row. All other comparisons were significant at p < 0.05*.

For the predictors, male teachers were less likely to correspond to the two types of *Flourisher* profiles and to the *Job-Resourced-Average* profile than to the *Balanced-Average* or *Low-Resourced-Struggler* profiles. Teachers with greater teaching experience were more likely to correspond to the *Low-Demand-Flourisher* profile than all other profiles. Teachers with greater teaching experience were also more likely to correspond to the *Mixed-Demand-Flourisher, Job-Resourced-Average*, and the *Balanced-Average* profiles than to the two types of *Struggler* profiles. Taken together, these results suggest that male teachers and less experienced teachers were more likely to be in the arguably less desirable profiles.

For the outcomes, members of the *Mixed-Demand-Flourisher* profile displayed the highest levels of job satisfaction and occupational commitment, followed by members of the *Low-Demand-Flourisher* profile. The next highest level was observed in the *Job-Resourced-Average* profile, followed equally by the *Balanced-Average* profile and *Mixed-Resourced-Struggler* profile. Finally, the *Low-Resourced-Struggler* profile displayed the lowest levels on both outcomes. For the outcomes after correcting for the predictors, the pattern of results was the same, with one exception: the *Mixed-Demand-Flourisher* and the *Low-Demand-Flourisher* exhibited similar levels of occupational commitment after controlling for teachers' background characteristics.

### Phase 1: Multilevel LPA

#### Profile Identification and Description

Model fit statistics for the multilevel solutions with 1 through 8 profiles estimated separately in Australia and England are reported in the bottom section of Table 2 and corresponding elbow plots are reported in Supplementary Figure 2. For Australia, the 3 profile L2 solution resulted in the lowest value for the CAIC, BIC, and SSA-BIC, whereas the AIC continued to decrease up to 8 profiles. However, the elbow plot showed a flattening in the decrease of the value of the AIC and SSA-BIC around three profiles, although this flattening was first apparent in relation to the 2 profile solution for the CAIC and BIC. For England, the 3 profile L2 solution resulted in the lowest value of the AIC, whereas the 2 profile solution resulted in the lowest values for the CAIC, BIC, and SSA-BIC. For the AIC, the elbow plot showed a flattening at two profiles. In summary, these statistics generally suggested than the optimal solution should have 2 or 3 profiles. These two solutions were thus further examined for conceptual relevance, parsimony, and meaningfulness. This examination revealed that the profile added as part of the 3 profile solutions did not meaningfully differ from the profiles already included in the 2 profile solution in either country. Accordingly, a solution comprising 2 school-level profiles was retained in both countries.

**Figure 3:**
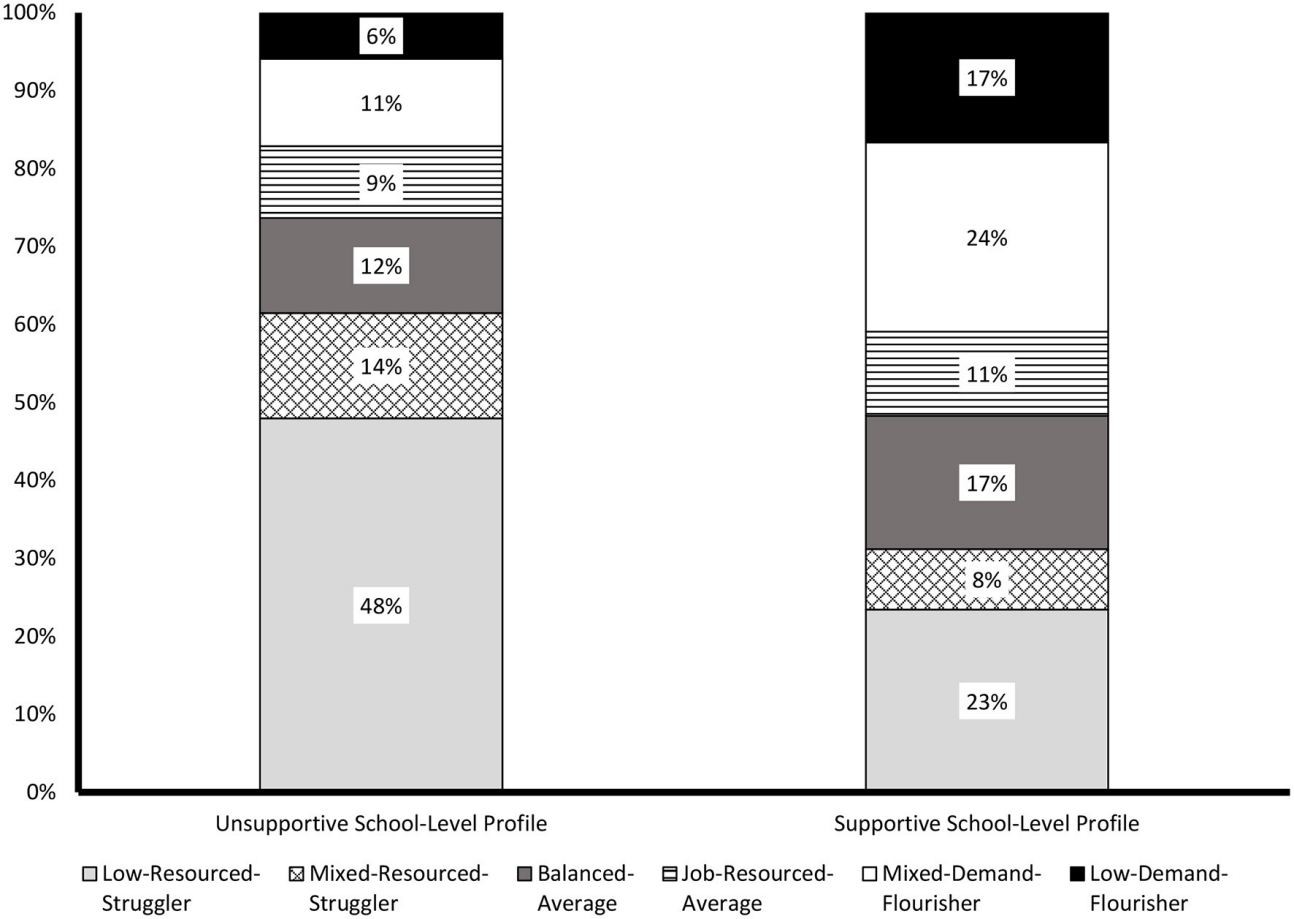
Multilevel LPA results (with L1 and L2 distributional constraints) showing the school-level profiles for both countries.

The results from the L2 tests of profile similarity conducted across the two countries are reported in the bottom section of Table 3, and support the complete similarity (configural, structural, and distributional) of the solution across Australia and England. A graphical representation of the final 2 profile solution of L2-distributional similarity is presented in Figure 3. Examination of this solution suggested the presence of an Unsupportive school profile (43% of the schools) and a Supportive school profile (57% of the schools). The *Unsupportive school profile* included a high proportion of members from the *Low-Resourced-Struggler* (48%) profile, followed by the *Mixed-Resourced-Struggler* (14%), *Balanced-Average* (12%), *Mixed-Demand-Flourisher* (11%), *Job-Resourced-Average* (9%), and *Low-Demand-Flourisher* (6%) profiles. Turning to the second school profile, the *Supportive school profile* included a high proportion of the *Mixed-Demand-Flourisher* (24%), followed by the *Low-Resourced-Struggler* (23%), the *Low-Demand-Flourisher* and *Balanced-Average* profiles (both 17%), the *Job-Resourced-Average* (11%), and finally the *Mixed-Resourced-Struggler* (8%).

#### Profile Prediction and Outcomes

The results from the tests of predictive (associations with predictors), explanatory (associations with outcomes), and adjusted-explanatory (associations with outcomes controlling for predictors) similarity are shown in Table 3. These results support the equivalence of the associations between the profiles and their predictors across countries, but revealed differences across countries related to the associations between the profiles and the outcomes (with and without controls). The results from these analyses are reported in Table 6 for the predictors, and in Table 7 for the outcomes and adjusted outcomes. For the predictors, the results reveal a single statistically significant association suggesting that schools with a higher proportion of low-SES students were more likely to correspond to the *Unsupportive* school profile than to the *Supportive* one.

**Table 6:** The role of school characteristics in predicting membership in the supportive school profile in both countries (Multilevel LPA).

	***b***	***SE***	***OR***
School location	0.510	0.475	1.665
School size	0.070	0.186	1.073
Proportion students from low-SES backgrounds	−1.054^**^	0.249	0.349
Proportion students from NESB backgrounds	0.380	0.272	1.462

** *p ≤ 0.01*;

*b, multinomial logistic regression coefficient; SE, standard error of the coefficient; OR, odds ratio; SES, socio-economic-status; NESB, non-English speaking backgrounds. For school location and school size, higher values represent more populous locations and larger schools, respectively. For proportion of students from low-SES or NESB backgrounds, larger values represent higher proportions in a school*.

**Table 7:** Means of school-level outcomes from explanatory similarity test and adjusted-explanatory similarity test (Multilevel LPA).

	**Unsupportive School Profile** ***M*** **(95% CI)**		**Supportive School Profile** ***M*** **(95% CI)**	
	**Australia**	**England**	**Australia**	**England**
**Explanatory similarity test**				
School-average job satisfaction	−0.114 (−0.158, −0.070)	−0.233 (−0.276, −0.190)	0.148 (0.094, 0.203)	0.055 (0.028, 0.083)
School-average occupational commitment	−0.039 (−0.062, −0.016)	−0.203 (−0.233, −0.173)	0.109 (0.068, 0.150)	−0.027 (−0.047, −0.007)
**Adjusted-explanatory similarity test (with controls for predictors)**				
School-average job satisfaction	−0.055 (−0.154, 0.044)	−0.162 (−0.268, −0.057)	0.140 (0.062, 0.217)	0.075 (−0.010, 0.160)
School-average occupational commitment	0.014 (−0.043, 0.072)	−0.135 (−0.202, −0.068)	0.117 (0.063, 0.172)	−0.007 (−0.062, 0.048)

*All comparisons within and across country were significantly different at p < 0.05 for both tests*.

For the outcomes, due to the lack of explanatory similarity across country, we compared the school-level means of the teacher outcomes within and across Australia and England. In both countries, the *Supportive* school profile displayed significantly higher school-average job satisfaction and occupational commitment than the *Unsupportive* one. When comparing matched profiles across the two countries, the two school profiles from the Australian sample displayed significantly higher levels on the two outcomes than the matching profiles estimated in the English sample. For the outcomes adjusted for school characteristics, the pattern of results was the same as that found for the unadjusted outcomes.

### Phase 2: School Profiles and Instructional Support and School-Average Achievement

Phase 2 involved examining the extent to which the school-level profiles are associated with student outcomes (i.e., perceived instructional support and academic achievement). An initial model was estimated including the four school characteristics as predictors of school profile membership. As before, the results from the multinomial logistic regressions revealed only one statistically significant association, showing that schools with a higher proportion of low-SES students were more likely to correspond to the *Unsupportive* profile than to the *Supportive* one (*b* = 1.69, *SE* = 0.74, *p* = 0.02, odds ratio = 5.44).

Next, the associations among the profiles and the school-average outcomes were examined. These results are presented in Table 8 and first reveal that the *Supportive* school profile was characterized by significantly higher levels of school-average autonomy-support, instrumental help, and teacher warmth when compared to the *Unsupportive* school profile. The *Supportive* school profile was also characterized by significantly higher school-average levels of reading, mathematics, and science achievement than the *Unsupportive* school profile. Finally, these associations were re-estimated while controlling for the school characteristics (Table 8). The results from these analyses where identical to those previously discussed without including this control.

**Table 8:** Means of student-reported outcomes related to instructional practice and achievement (Phase 2).

	**Unsupportive school profile** ***M* (95% CI)**	**Supportive school profile** ***M* (95% CI)**	***p*-value**
**Model (with no covariate controls)**			
***Instructional support***			
Autonomy-support	−0.052 (−0.078, −0.026)	0.043 (0.019, 0.068)	<0.01
Instrumental help	−0.032 (−0.056, −0.008)	0.027 (0.004, 0.050)	<0.01
Teacher warmth	−0.077 (−0.115, −0.038)	0.064 (0.027, 0.100)	<0.01
**School-average achievement**			
Reading achievement	456 (443, 470)	531 (519, 544)	<0.01
Mathematics achievement	452 (443, 462)	515 (503, 528)	<0.01
Science achievement	462 (51, 473)	530 (518, 543)	<0.01
**Adjusted model (with covariate controls)**			
***Instructional support***			
Autonomy-support	−0.057 (−0.077, −0.036)	0.046 (0.030, 0.061)	<0.01
Instrumental help	−0.073 (−0.101, −0.036)	0.001 (−0.010, 0.011)	<0.01
Teacher warmth	−0.046 (−0.065, −0.028)	0.094 (0.070, 118)	<0.01
**School-average achievement**			
Reading achievement	498 (487, 508)	537 (529, 545)	<0.01
Mathematics achievement	478 (461, 495)	511 (496, 526)	<0.01
Science achievement	503 (484, 523)	539 (521, 557)	<0.01

*The achievement values are reported as whole numbers as per OECD guidelines (OECD, 2019b). For comparison, OECD means and SD for the broader Australian sample that participated in PISA 2018 are, respectively, 503 and 105 for reading achievement, 491 and 85 for mathematics achievement, and 503 and 95 for science achievement. The instructional support factors were directly estimated in standardized units across the whole sample with M ≈ 0, SD = 0.094 for autonomy-support, SD = 0.089 for instrumental help, and SD = 0.135 for teacher warmth*.

## Discussion

Our study was designed to replicate and extend Collie et al.'s (2020a) research. In Phase 1 (the replication phase), many of the findings obtained by Collie et al. (2020a) were reproduced. In particular, our results led to the identification of the same set of school profiles (a Supportive school profile and an Unsupportive school profile), and revealed a generally similar pattern of associations with predictors and outcomes at the teacher and school level. However, one notable difference was the identification of six, rather than five, teacher profiles: Low-Demand-Flourisher, Mixed-Demand-Flourisher, Job-Resourced-Average, Balanced-Average, Mixed-Resourced-Struggler, and Low-Resourced-Struggler. More precisely, the current results led to the identification of five profiles matching those identified by Collie et al. (2020a), along with the identification of one additional Struggler profile (Mixed-Resourced-Struggler) not previously identified. Importantly, these six profiles were identical in both the Australian and English samples. In Phase 2 (the extension phase, conducted among a subsample of Australian teachers and schools), our results revealed that schools including a greater proportion of low-SES students were more likely to correspond to the Unsupportive school profile. In addition, the Supportive school profile was found to be associated with significantly higher school-average levels of autonomy-support, instrumental help, warmth, and achievement in reading, mathematics, and science. Key findings and implications are discussed below. Because many of the findings from Collie et al. (2020a) were replicated, we focus on the findings that are different, along with those that extend prior work.

### Findings of Note From Single-Level Analyses in Phase 1

Six teacher profiles, identical across Australia and England, were identified in this study. Four of these profiles replicated Collie et al.'s (2020a) results (i.e., Low-Demand-Flourisher, Mixed-Demand-Flourisher, Job-Resourced-Average, and Balanced-Average profiles). Collie et al.'s (2020a) Struggler profile was also identified in the present study, but had to be re-labeled the Low-Resource-Struggler profile to reflect the fact that the present results revealed one additional struggler profile, labeled the Mixed-Resourced-Struggler profile. This new profile was characterized by high job demands, mixed job resources (average teacher collaboration, high input), and low self-efficacy. In that sense, this new profile was similar to the Low-Resourced-Struggler profile in some regards (both characterized by high demands and low self-efficacy), but differed by displaying a rather high (vs. low) level of input in decision-making and a close to average (vs. very low) level of teacher collaboration.

It is possible that this new profile might have emerged as a result of the changes to the nature of teachers' work that have occurred between 2013 and 2018. For example, recent policy priorities have highlighted the need to better support teachers (OECD, 2019a), which might have enabled some of the more at-risk teachers to have greater input at work, and to benefit from slightly improved collaboration opportunities. Indeed, there have been efforts to seek teachers' perspectives on workload and to reduce unnecessary tasks (Higton et al., 2017; OECD, 2019a), which might have been particularly helpful to at least a subset of the teachers who struggled the most with their job demands. At the same time, the complexity of teachers' work has also received greater acknowledgment recently (Guerriero and Révai, 2017). Thus, the Mixed-Resourced-Struggler profile might both reflect the increased awareness of the need to support teachers and to involve them in decision making, but also the growing pressures of the job. Future research, including qualitative and mixed methods approaches, will be helpful to better understand the contingencies at play in the emergence of this new profile.

In relation to the relative prevalence of the identified profiles, it is interesting to consider differences across our study and prior results. Collie et al. (2020a) found that the two Flourisher profiles represented about one-third of their combined sample, the two Average profiles represented about one-half, and the Struggler profile represented one-fifth. In our study, the proportions were similar for the two Flourishers profiles (about one-quarter). However, the proportions were the opposite to those found by Collie et al. (2020a) for the two Average profiles (about one-quarter, rather than one-half) and for the two Struggler profiles (about one-half, rather than one fifth). Thus, although the profiles were similar in nature across the two studies, their prevalence differed greatly. Perhaps these shifts may be explained by some of the policy and practice changes that occurred between 2013 and 2018. For example, increases in compliance, accountability, and external evaluations across both countries between 2013 and 2018 (OECD, 2019a) may have meant teachers had less time and energy for professional development, managing disruptive behavior, and collaborating with colleagues. These changes, in turn, may have led to the greater prevalence of the two Struggler profiles in 2018. Compliance and accountability are also known to be negatively associated with reduced self-efficacy among teachers (von der Embse et al., 2016). Taken together, a key strength of the current study was the use of a replication sample collected 5 years after the original study. More precisely, it was because we conducted replication with nationally-representative data that we were able to identify teacher (and school) demand-resource profiles and then consider how macro-level changes to policy and practice may be implicated in the workplace experiences of distinct subpopulations of teachers. Going forward, it will be important to extend this knowledge by collecting data from the same teachers longitudinally to test whether teachers move between profiles over time, and what initiatives help teachers to move into more adaptive profiles.

The results involving the predictors and outcomes at the teacher-level were largely the same as those reported by Collie et al. (2020a), with the exception that, in addition to obtaining predictive similarity (i.e., evidence that predictions were the same across counties) like Collie et al. (2020a), we also obtained evidence of explanatory and adjusted-explanatory similarity (i.e., evidence that associations with outcomes where the same across countries before, and after controlling for school characteristics). Thus, the Mixed-Demand-Flourisher typically displayed the highest levels of job satisfaction and commitment, while the Low-Resourced-Struggler displayed the lowest levels. In practical terms, these results suggest that there might be merit in adopting a broad focus on reducing demands and increasing resources. This could involve the development of professional learning communities to help teachers build positive collaboration and self-efficacy (e.g., Durksen et al., 2017). Inviting teachers to have a say in school-level decisions and actively listening to teachers' perspectives and needs may be helpful for building their input in decision-making (see Collie et al., 2020a for additional implications for practice).

### Findings of Note From Multilevel Analyses in Phase 1

Like Collie et al.'s (2020a), the present study led to the identification of a Supportive school profile and of an Unsupportive school profile that were equivalent across Australia and England. The results related to the outcomes of these profiles at the school level also generally match those reported, and discussed, by Collie et al. (2020a). For this reason, we focus on several novel findings related to the predictors of membership into these profiles at the school level. Notably, our results involving predictors provide the first source of information regarding associations between school characteristics and school demand-resource profiles. More precisely, our results showed that there was equivalence in the way these predictors were associated with the school profiles across the two countries. Thus, in Australia and England, schools with a higher proportion of low-SES students were more likely to correspond to the Unsupportive school profile than to the Supportive one. This finding is, unfortunately, unsurprising given that schools serving disadvantaged students often face many additional challenges and are often under resourced (Thomson et al., 2020), which is likely to result in higher demands and fewer resources for teachers. Going forward, it will be important to examine school funding and resourcing alongside SES to disentangle their different roles. More broadly, funding cuts and austerity measures that have occurred over the past few years to services beyond school (e.g., services for disadvantaged families) have also likely increased the challenges for teachers and schools serving more disadvantaged students (e.g., Hanley et al., 2020). These broader societal impacts are important to consider in future research. Notably, school location, school size, and the proportion of students from non-English speaking backgrounds did not significantly predict the school profiles. Thus, beyond the role of SES, these other factors were not found to be associated with demand-resource profiles.

### Findings of Note Involving Student Outcomes in Phase 2

Our final set of findings involved associations between the school profiles and the student outcomes, which has not been examined in prior research. More precisely, these results showed that, when compared to the Unsupportive school profile, the Supportive school profile tended to be associated with higher levels of school-average autonomy-support, instrumental help, and warmth, as well with higher levels of student achievement (across all three indicators of achievement considered in this study). These findings provide new evidence that demand-resource profiles matter not only for teachers' outcomes, but also for students' outcomes. The results extend prior work, which has demonstrated that teacher personal resource profiles tended to be associated with instructional support at the classroom-level (Klusmann et al., 2008), by showing that these associations are also salient at the school-level. Indeed, both the prevalence and nature of different teacher profiles within a school are associated with student outcomes, and this occurs even after controlling for school characteristics like SES. It is possible that these findings occurred because schools in which a greater number of teachers experience positive relationships at work, experience greater support at work, and are more confident in their teaching are likely to afford a more supportive and effective learning environment for students (e.g., Burić and Kim, 2020). More precisely, when teachers at a school feel supported and confident, they are more likely to create an environment where students feel better supported and are enriched in their learning (Collie et al., 2020a). In practical terms, these findings highlight the importance of creating supportive working climates not only for teachers, but also for students.

### Limitations

Several limitations are important to consider when interpreting our study's findings. First, although the use of TALIS 2018 data comes with significant strengths (adequate sample size to conduct multilevel modeling, nationally representative data), it remains cross-sectional in nature. This means that we were not able to test for the directionality of any of the associations between the profiles and the outcomes. Importantly, our study was grounded in theory, which provided support for our hypotheses. Notwithstanding this, going forward it will be interesting for longitudinal and intervention research to examine the extent to which there may be reciprocal relations among the profiles and outcomes. Second, a strength of our study was that it involved data from both teachers and students. However, the TALIS-PISA link does not allow us to match students and teachers at the classroom-level. Thus, we cannot know whether the students who participated in PISA were taught by the teachers who participated in TALIS. Importantly though, all the students and teachers in our study were members of the same school—and that is where we focused in linking students and teachers (i.e., at the school-level). In future, it will be worth expanding this to consider links at the classroom-level, and also to include principal data about the job demands and resources present in the school. Given that the TALIS-PISA 2018 link was not conducted for England, it will also be important to test whether and how the links between the profiles and the student outcomes can be replicated in England, as well as in other countries. Third, one potential criticism of person-centered analysis is whether profiles are idiosyncratic to a particular sample. Our analytic approach has several important strengths that help to address this concern: profiles were examined (and found to be equivalent) across two countries and also largely replicated prior research (Collie et al., 2020a). Other strengths of our approach were that profiles were examined at two levels, and predictors and outcomes were entered after profile identification (and thus did not influence the profile solutions). Moreover, our models allowed us to examine important theoretical moderating mechanisms in more complex ways than possible in variable-centered modeling. Notwithstanding these strength, additional research (including more replication) is needed to provide additional evidence about the generalizability of our profiles. Because our study was intended to act as a replication and an extension, we focused on Australia and England to align with the Collie et al. (2020a) study. Given that the demands and resources examined in the present study are also experienced by teachers worldwide, in future it will be important to ascertain whether similar profiles can be identified in other countries as well.

## Conclusion

The aim of the present study was to conduct a replication and extension of prior research examining demand-resource profiles among teachers and schools. Many of the findings from Collie et al. (2020a) were replicated, providing important support for the profiles and their relevance to teachers in Australia and England, and potentially in other contexts. In addition, several novel findings help to advance knowledge about the role of demands and resources in teachers' work and how these are linked not only with teachers' outcomes, but also students' outcomes. We speculate that some notable changes experienced within educational systems between these two studies might have had an impact on the current results, thus highlighting the importance of monitoring the impact of political and administrative changes for different educational systems worldwide. Moreover, our results make it clear that teachers should be supported at work. Not only is this important for the individual teacher, it is relevant for shaping the working and learning climate across a whole school.

## Data Availability Statement

Publicly available datasets were analyzed in this study. This data can be found here: https://www.oecd.org/education/talis/.

## Ethics Statement

The studies involving human participants were reviewed and approved by the UNSW Human Research Ethics Committee. Written informed consent for participation was not required for this study in accordance with the national legislation and the institutional requirements.

## Author Contributions

All authors listed have made a substantial, direct and intellectual contribution to the work, and approved it for publication.

## Conflict of Interest

The authors declare that the research was conducted in the absence of any commercial or financial relationships that could be construed as a potential conflict of interest.

## Publisher's Note

All claims expressed in this article are solely those of the authors and do not necessarily represent those of their affiliated organizations, or those of the publisher, the editors and the reviewers. Any product that may be evaluated in this article, or claim that may be made by its manufacturer, is not guaranteed or endorsed by the publisher.

## Footnotes

^1^Coefficient omega was calculated from the most invariant model in the measurement invariance tests and thus reflects the reliability across both countries.
