# Supplementary material for: A Multilevel Person-Centered Examination of Teachers' Workplace Experiences: Replication and Extension With Links to Instructional Support and Achievement
Source: Front Psychol. 2021 Aug 6;12:711173. doi: 10.3389/fpsyg.2021.711173 (PMC8377360; doi:10.3389/fpsyg.2021.711173)
Supplement: Supplementary file 1 [file Data_Sheet_1.docx]

**Supplemental Materials for**

**A Multilevel Person-Centered Examination of Teachers’ Workplace Experiences:**

**Replication and Extension with Links to Instructional Support and Achievement**

**Preliminary Analyses: Measurement Invariance Testing**

**Specifications**

The first set of measurement invariance tests involved the profile indicator variables measured at the teacher-level only. In these models, separate factors were specified for each demand and resource, including the three dimensions of self-efficacy (i.e., self-efficacy for student engagement, self-efficacy for classroom management, and self-efficacy for instruction). These three subdimension of self-efficacy were then allowed to define a higher-order factor representing global levels of self-efficacy. The second set of measurement invariance tests involved the teacher outcomes, modelled using a multilevel-CFA at the teacher- and school-level. Finally, the third set of tests involved the instructional support outcomes, modelled using a multilevel CFA at the student- and school-level. Tests of measurement invariance (Millsap, 2011) were estimated in the following sequence to ensure that the ratings obtained in the Australian and English samples could be considered comparable: (a) configural (same measurement model), (b) weak (same factor loadings), (c) strong (same intercepts), (d) strict (same residuals), (e) latent variance-covariance, and (f) latent means.

For the profile indicator measurement model, tests of measurement invariance were first conducted with the single-order factors of self-efficacy (Cheung, 2008). The higher-order self-efficacy factor was then added and its parameters were progressively constrained across country using the same steps shown above. In this model, all other factors that had been tested in the models involving the single-order factors of self-efficacy were constrained up to latent mean invariance and the first-order self-efficacy factors were constrained up to the strict invariance model. This provided an invariance test of the higher-order factor.

For the multilevel-CFA model involving teacher’s outcomes, tests of isomorphisms (equality of factor loadings across levels) were incorporated between tests of weak and strong invariance. Finally, for the multilevel-CFA model involving the instructional support outcomes, no tests of measurement invariance could be run (these outcomes are only available in Australia), but we still verified the measurement isomorphism of the model across levels. Model fit was assessed using the chi-square test of exact fit (χ^2^), the Root Mean Square Error of Approximation (RMSEA), the Comparative fit Index (CFI), and the Tucker-Lewis Index, with values ≥ .90 and .95 on the CFI/TLI, and ≤ .08 or .06 on the RMSEA, respectively indicating adequate and excellent model fit (Hu & Bentler, 1999; Marsh et al., 2005). Invariance was considered to be established as long as RMSEA values did not increase by more than .015, and CFI/TLI values did not increases by more than .010 between a model and the previous one in the sequence (Chen, 2007; Cheung & Renvold, 2002; Marsh et al., 2005).

**Results**

The fit indices obtained for these preliminary analyses are reported in Table S1 of these online supplements. All models achieved an acceptable to excellent level of model fit. For the profile indicator variables, the results supported the complete measurement invariance of the first- and higher- order structure of these factors across the two countries (all ΔRMSEA ≤ .015; all ΔCFI/TLI ≤ .01). The latent correlations and other parameter estimates from this final measurement model are reported in Table S2a and Table S3 of these online supplements. These results reveal well-differentiated and well-defined factors. For the teacher outcomes, the results also supported the complete measurement invariance (and measurement isomorphism) of the multilevel-CFA solution across the two countries. The latent correlations and other parameter estimates obtained from this final model are reported in Table S2b and Table S4, and reveal well-defined factors. Finally, for the instructional support outcomes, the results also supported the measurement isomorphism of the multilevel-CFA solution. The latent correlations and other parameter estimates obtained from this final model are reported in Table S2c and Table S5 and reveal well-defined factors.

**References Used in this Supplement**

Chen, F.F. (2007). Sensitivity of goodness of fit indexes to lack of measurement invariance. *Structural Equation Modeling*, *14*, 464–504.

Cheung, G.W. (2008). Testing equivalence in the structure, means, and variances of higher-order constructs with structural equation modeling. *Organizational Research Methods*, *11*, 593-613.

Cheung, G. W. & Rensvold, R. B. (2002). Evaluating goodness-of fit indexes for testing measurement invariance. *Structural Equation Modeling*, *9*, 233–255.

Hu, L.T., & Bentler, P.M. (1999). Cutoff criteria for fit indexes in covariance structure analysis: Conventional criteria versus new alternatives. *Structural Equation Modeling*, *6*, 1-55.

Marsh, H.W., Hau, K.-T., & Grayson, D. (2005). Goodness of fit evaluation in structural equation modeling. In A. Maydeu-Olivares & J. McArdle (Eds.), *Contemporary Psychometric* (pp.275-340). Erlbaum.

Millsap, R. (2011). *Statistical Approaches to Measurement Invariance*. Taylor & Francis.

**Table S1**

*Measurement Invariance Tests*

|  | χ^2^ | *df* | RMSEA | CFI | TLI | Δχ^2^ | Δdf | ΔRMSEA | ΔCFI | ΔTLI |
| --- | --- | --- | --- | --- | --- | --- | --- | --- | --- | --- |
| *Profile indicator variables* |  |  |  |  |  |  |  |  |  |  |
| *Model with first-order self-efficacy factors* | | | | | | | | | | |
| Configural | 3816.333* | 606 | .037 | .936 | .926 | — | — | — | — | — |
| Weak | 3869.959* | 626 | .037 | .937 | .928 | 52.186 | 20 | .000 | .001 | .002 |
| Strong | 4058.436* | 646 | .037 | .932 | .926 | 187.196 | 20 | .000 | -.005 | -.002 |
| Strict | 4226.184* | 673 | .037 | .929 | .926 | 168.051 | 27 | .000 | -.003 | .000 |
| Latent Variance-Covariance | 4294.921* | 701 | .037 | .929 | .928 | 67.405 | 28 | .000 | .000 | .002 |
| Latent Means | 4491.882* | 708 | .037 | .925 | .925 | 158.593 | 7 | .000 | -.004 | -.003 |
| *Model with higher-order self-efficacy factors* | | | | | | | | | | |
| Configural | 4517.132* | 703 | .038 | .924 | .924 | — | — | — | — | — |
| Weak | 4518.906* | 705 | .038 | .924 | .925 | 2.470 | 2 | .000 | .000 | .001 |
| Strong | 4518.111* | 707 | .038 | .924 | .925 | 1.049 | 2 | .000 | .000 | .000 |
| Strict | 4526.277* | 710 | .037 | .924 | .925 | 8.328 | 3 | -.001 | .000 | .000 |
| Latent Variance-Covariance | 4536.711* | 715 | .037 | .924 | .925 | 7.077 | 5 | .000 | .000 | .000 |
| Latent Means | 4605.816* | 716 | .038 | .923 | .924 | 92.646 | 1 | .001 | -.001 | -.001 |
| *Teacher Outcomes* |  |  |  |  |  |  |  |  |  |  |
| Configural | 1114.090* | 52 | .075 | .934 | .894 | — | — | — | — | — |
| Weak | 1116.871* | 62 | .068 | .935 | .912 | 60.396 | 10 | -.007 | .001 | .018 |
| Weak with isomorphic constraints | 1160.465* | 67 | .067 | .932 | .915 | 52.112 | 5 | -.001 | -.003 | .003 |
| Strong | 1262.429* | 72 | .067 | .926 | .914 | 106.528 | 5 | .000 | -.006 | -.001 |
| Strict | 1194.080* | 86 | .059 | .931 | .933 | 44.833 | 14 | -.008 | .005 | .019 |
| Latent Variance-Covariance | 1196.659* | 92 | .057 | .932 | .938 | 25.067 | 6 | -.002 | .001 | .005 |
| Latent Means | 1318.584* | 94 | .060 | .924 | .932 | 119.240 | 2 | .003 | -.008 | -.006 |
| *Instructional support outcomes* |  |  |  |  |  |  |  |  |  |  |
| No isomorphic constraints | 265.219* | 64 | .039 | .985 | .978 | — | — | — | — | — |
| Isomorphic constraints | 223.177* | 71 | .032 | .988 | .985 | 13.278 | 7 | -.007 | .003 | .007 |

*Note*. * p ≤ .01; χ^2^ = robust chi-square test of exact fit; *df* = degrees of freedom; RMSEA = root mean square error of approximation; CFI = comparative fit index; TLI = Tucker-Lewis Index; Δ = change from the previous model.

**Table S2a**

*Latent Correlations from the Latent Mean Invariance Model Involving Profile Indicator Variables*

|  | 1. | 2. | 3. | 4. |
| --- | --- | --- | --- | --- |
| 1. Barriers to professional learning |  |  |  |  |
| 1. Disruptive student behavior | .220** |  |  |  |
| 1. Teacher collaboration | -.239** | -.089** |  |  |
| 1. Teacher input | -.470** | -.176** | .293** |  |
| 1. Teacher self-efficacy | -.173** | -.412** | .247** | .181** |

*Note.* * *p* < .05, ** *p* < .01.

**Table S2b**

*Latent Correlations from the Latent Mean Invariance Model Involving Teacher Outcomes*

|  | Teacher-level |  | School-level |
| --- | --- | --- | --- |
| Job satisfaction with occupational commitment | .695** |  | .762** |

*Note.* * *p* < .05, ** *p* < .01.

**Table S2c**

*Latent Correlations from the Isomorphic Invariance Model Involving Student Outcomes*

|  | Autonomy-support | Instrumental help |
| --- | --- | --- |
| *Student-level* |  |  |
| Autonomy-support |  |  |
| Instrumental help | .421** |  |
| Teacher warmth | .555** | .475** |
| *School-level* |  |  |
| Autonomy-support |  |  |
| Instrumental help | .785** |  |
| Teacher warmth | .889** | .451** |

*Note.* * *p* < .05, ** *p* < .01.

**Table S3**

*Standardized Factor Loadings (λ) and Residuals (δ) from the Latent Mean Invariance Model Involving Profile Indicator Variables*

|  | Model with first order self-efficacy | |  | Model with higher-order self-efficacy | |
| --- | --- | --- | --- | --- | --- |
|  | λ | δ |  | λ | δ |
| *Barriers to PL* |  |  |  |  |  |
| TT3G28B | .575 | .669 |  | .575 | .669 |
| TT3G28C | .701 | .508 |  | .702 | .507 |
| TT3G28D | .572 | .673 |  | .571 | .673 |
| TT3G28E | .356 | .873 |  | .356 | .873 |
| TT3G28F | .567 | .678 |  | .567 | .678 |
| TT3G28G | .699 | .511 |  | .699 | .512 |
| *Disruptive student behavior* |  |  |  |  |  |
| TT3G41A | .800 | .359 |  | .800 | .360 |
| TT3G41C | .915 | .164 |  | .914 | .164 |
| TT3G41D | .907 | .177 |  | .908 | .176 |
| *Teacher Collaboration* |  |  |  |  |  |
| TT3G33D | .630 | .603 |  | .629 | .604 |
| TT3G33E | .727 | .471 |  | .727 | .471 |
| TT3G33F | .677 | .542 |  | .678 | .540 |
| *Teacher input* |  |  |  |  |  |
| TT3G48A | .732 | .465 |  | .732 | .464 |
| TT3G48D | .851 | .276 |  | .851 | .276 |
| TT3G48E | .801 | .358 |  | .801 | .358 |
| *Self-efficacy for student engagement* |  |  |  |  |  |
| TT3G34A | .829 | .313 |  | .829 | .313 |
| TT3G34B | .857 | .266 |  | .856 | .268 |
| TT3G34E | .689 | .526 |  | .690 | .523 |
| TT3G34G | .602 | .638 |  | .602 | .638 |
| *Self-efficacy for classroom management* |  |  |  |  |  |
| TT3G34D | .796 | .366 |  | .797 | .365 |
| TT3G34F | .706 | .502 |  | .706 | .502 |
| TT3G34H | .819 | .329 |  | .818 | .331 |
| TT3G34I | .760 | .422 |  | .762 | .420 |
| *Self-efficacy for instruction* |  |  |  |  |  |
| TT3G34C | .616 | .620 |  | .615 | .622 |
| TT3G34J | .668 | .554 |  | .668 | .553 |
| TT3G34K | .704 | .504 |  | .704 | .505 |
| TT3G34L | .731 | .466 |  | .732 | .465 |
| *Self-efficacy (higher-order factor)* |  |  |  |  |  |
| Self-efficacy for student engagement | — | — |  | .794 | .370 |
| Self-efficacy for classroom management | — | — |  | .759 | .424 |
| Self-efficacy for instruction |  |  |  | .789 | .378 |

*Note*. Barriers to PL = Barriers to professional learning.

**Table S4**

*Standardized Factor Loadings (λ) and Residuals (δ) from the Multilevel Latent Mean Invariance Model Involving Teacher Outcomes*

|  | *λ* | *δ* |
| --- | --- | --- |
| *Teacher-level* |  |  |
| *Job satisfaction* |  |  |
| TT3G53E | .825 | .320 |
| TT3G53G | .783 | .386 |
| TT3G53J | .756 | .429 |
| *Occupational commitment* |  |  |
| TT3G53A | .736 | .459 |
| TT3G53B | .828 | .314 |
| TT3G53DR | .733 | .463 |
| TT3G53FR | .751 | .436 |
| *School-level* |  |  |
| *Job satisfaction* |  |  |
| TT3G53E | 1.000 | .000 |
| TT3G53G | .851 | .276 |
| TT3G53J | .931 | .134 |
| *Occupational commitment* |  |  |
| TT3G53A | .986 | .028 |
| TT3G53B | 1.000 | .000 |
| TT3G53DR | .956 | .086 |
| TT3G53FR | .971 | .058 |

**Table S5**

*Standardized Factor Loadings (λ) and Residuals (δ) from the Multilevel Model Involving Instructional Support Outcomes with Isomorphic Constraints*

|  | *λ* | *δ* |
| --- | --- | --- |
| *Student-level* |  |  |
| *Autonomy-support* |  |  |
| ST211Q01HA | .830 | .312 |
| ST211Q02HA | .879 | .227 |
| ST211Q03HA | .902 | .186 |
| *Instrument help* |  |  |
| ST104Q02NA | .829 | .312 |
| ST104Q03NA | .897 | .195 |
| ST104Q04NA | .916 | .161 |
| *Teacher warmth* |  |  |
| ST213Q01HA | .837 | .299 |
| ST213Q02HA | .779 | .394 |
| ST213Q03HA | .770 | .407 |
| ST213Q04HA | .865 | .252 |
| *School-level* |  |  |
| *Autonomy-support* |  |  |
| ST211Q01HA | .996 | .009 |
| ST211Q02HA | .999 | .002 |
| ST211Q03HA | .961 | .076 |
| *Instrumental help* |  |  |
| ST104Q02NA | .924 | .146 |
| ST104Q03NA | .984 | .031 |
| ST104Q04NA | 1.000 | .000 |
| *Teacher warmth* |  |  |
| ST213Q01HA | 1.000 | .000 |
| ST213Q02HA | .961 | .076 |
| ST213Q03HA | .942 | .113 |
| ST213Q04HA | .970 | .058 |

**Figure S1**

*Elbow Plots for Single-level LPA Involving Australia (Figure S1a) and England (Figure S1b)*

a)

b)

**Figure S2**

*Elbow Plots for Multilevel LPA Involving Australia (Figure S2a) and England (Figure S2b)*

a)

b)

**Table S6**

*Teacher-Level Latent Profile Analysis Solution (Means and Variances) of Indicator Variables from Distributional Similarity Model*

|  | Profile indicator variables | | | | | | | | | | | | | |
| --- | --- | --- | --- | --- | --- | --- | --- | --- | --- | --- | --- | --- | --- | --- |
|  | Barriers to professional development | |  | Disruptive student behavior | |  | Teacher collaboration | |  | Teacher input | |  | Teacher self-efficacy | |
|  | *M*  (95% CI) | Variance  (95% CI) |  | *M*  (95% CI) | Variance  (95% CI) |  | *M*  (95% CI) | Variance  (95% CI) |  | *M*  (95% CI) | Variance  (95% CI) |  | *M*  (95% CI) | Variance  (95% CI) |
| Low-Demand- Flourisher | -.448  (-.536, -.360) | .815  (.742, .888) |  | -1.279  (-1.283, -1.276) | .001  (.001, .001) |  | .229  (.150, .308) | .529  (.423, .634) |  | .361  (.270, .451) | .773  (.664, .883) |  | .814  (.737, .891) | .274  (.220, .327) |
| Mixed-Demand- Flourisher | -.743  (-.860, -.626) | .649  (.548, .750) |  | -.162  (-.246, -.078) | .470  (.357, .583) |  | .590  (.513, .666) | .175  (.119, .232) |  | .647  (.541, .752) | .577  (.503, .650) |  | .461  (.366, .556) | .489  (.418, .560) |
| Job-Resourced-Average | -.299  (-.354, -.245) | .090  (.075, .105) |  | -.049  (-.140, .043) | .503  (.398, .607) |  | .449  (.375, .523) | .195  (.153, .237) |  | .370  (.364, .377) | .001  (.001, .001) |  | .189  (.100, .277) | .573  (.519, .627) |
| Balanced- Average | .096  (.024, .168) | .522  (.463, .581) |  | -.035  (-.040, -.013) | .001  (.001, .001) |  | -.012  (-.116, .093) | .516  (.407, .625) |  | -.089  (-.177, -.001) | .554  (.469, .640) |  | -.095  (-.188, -.002) | .323  (.284, .363) |
| Mixed-Resourced- Struggler | .286  (.212, .360) | .123  (.104, .143) |  | .401  (.318, .483) | .689  (.620, .757) |  | -.049  (-.146, .048) | .427  (.357, .497) |  | .297  (.289, .304) | .001  (.001, .001) |  | -.352  (-.445, -.259) | .691  (.627, .755) |
| Low-Resourced-Struggler | .584  (.518, .650) | .635  (.563, .707) |  | .491  (.433, .548) | .878  (.832, .942) |  | -.518  (-.576, -.460) | .915  (.863, .967) |  | -.669  (-.738, -.600) | .802  (.739, .865) |  | -.419  (-.467, -.372) | .768  (.722, .815) |

*Note.* 95% CI = 95% Confidence Interval.

**Mplus Syntax for L1 Profile Similarity Tests: Adjusted-Explanatory Similarity with Outcomes Freely Estimated Across Countries While Controlling for Predictors (Step 1 of 2)**

DATA: FILE IS "STDWEIGHTS.dat";

VARIABLE: NAMES ARE PLBARRS SMISBEH TCOLLAB INPUT TEFF TT3G01 TT3G11B WOCC WJS BOCC BJS SCHLOC NENRSTUD TC3G17A TC3G17C BAUTSUPP BCOMPSUP BTENTH PV1MATHM PV2MATHM PV3MATHM PV4MATHM PV5MATHM PV6MATHM PV7MATHM PV8MATHM PV9MATHM PV10MATH PV1READM PV2READM PV3READM PV4READM PV5READM PV6READM PV7READM PV8READM PV9READM PV10READ PV1SCIEM PV2SCIEM PV3SCIEM PV4SCIEM PV5SCIEM PV6SCIEM PV7SCIEM PV8SCIEM PV9SCIEM PV10SCIE IDPOP TCHWGT SCHWGT TEACHID IDCNTRY SCHLID;

USEVARIABLES = PLBARRS SMISBEH TCOLLAB INPUT TEFF TT3G01 TT3G11B WOCC WJS;

KNOWNCLASS = cg (IDCNTRY = 36 IDCNTRY = 926);

CLASSES = cg (2) c (6);

CLUSTER = SCHLID;

MISSING ARE *;

WEIGHT=TCHWGT;

ANALYSIS:

TYPE = MIXTURE COMPLEX;

STARTS = 0;

MODEL:

%OVERALL%

*!The predictors are regressed on the latent profile variable (C) and the outcomes. For the*

*!associations between the predictors and the latent profile variable (C), this means the*

*!associations were constrained to be equal across groups. If we had not attained Predictive*

*!similarity, these associations would have need to be freely estimated across groups.*

C ON TT3G01 TT3G11B;

*!The predictors are regressed on the outcomes so they serve as covariate controls (similar to ANCOVA).*

WOCC WJS ON TT3G01 TT3G11B;

*!Below, the means and variances for the different profiles are set to the parameters estimated*

*!in the L1-distributional similarity tests using the approach described in Collie et al. (2020).*

*!The labels (m1, m2) in parentheses can be deleted (given that the parameters are fixed) but*

*!they can also be retained in the analyses for greater simplicity (they are automatically*

*!included in Mplus SVALUES section of the output).*

*!Group 1 (Australia)*

%cg#1.c#1%

[ plbarrs@-0.44827 ] (m1);

[ smisbeh@-1.27932 ] (m2);

[ tcollab@0.22905 ] (m3);

[ input@0.36065 ] (m4);

[ teff@0.81401 ] (m5);

plbarrs@0.81505 (v1);

smisbeh@0.00059 (v2);

tcollab@0.52871 (v3);

input@0.77336 (v4);

teff@0.27371 (v5);

*!Parameter labels below refer to the mean levels of the outcome. In this syntax, the*

*!parameters are freely estimated across all profiles.*

[WJS] (js11);

[WOCC] (oc11);

%cg#1.c#2%

[ plbarrs@-0.74323 ] (m13);

[ smisbeh@-0.16230 ] (m14);

[ tcollab@0.58975 ] (m15);

[ input@0.64659 ] (m16);

[ teff@0.46102 ] (m17);

plbarrs@0.64896 (v13);

smisbeh@0.47020 (v14);

tcollab@0.17542 (v15);

input@0.57663 (v16);

teff@0.48903 (v17);

[WJS] (js12);

[WOCC] (oc12);

%cg#1.c#3%

[ plbarrs@-0.29924 ] (m30);

[ smisbeh@-0.04876 ] (m31);

[ tcollab@0.44911 ] (m32);

[ input@0.37034 ] (m33);

[ teff@0.18890 ] (m34);

plbarrs@0.09013 (v30);

smisbeh@0.50253 (v31);

tcollab@0.19470 (v32);

input@0.00056 (v33);

teff@0.57300 (v34);

[WJS] (js13);

[WOCC] (oc13);

%cg#1.c#4%

[ plbarrs@0.09603 ] (m19);

[ smisbeh@-0.03531 ] (m20);

[ tcollab@-0.01181 ] (m21);

[ input@-0.08906 ] (m22);

[ teff@-0.09510 ] (m23);

plbarrs@0.52200 (v19);

smisbeh@0.00059 (v20);

tcollab@0.51633 (v21);

input@0.55427 (v22);

teff@0.32349 (v23);

[WJS] (js14);

[WOCC] (oc14);

%cg#1.c#5%

[ plbarrs@0.28596 ] (m25);

[ smisbeh@0.40055 ] (m26);

[ tcollab@-0.04868 ] (m27);

[ input@0.29653 ] (m28);

[ teff@-0.35182 ] (m29);

plbarrs@0.12338 (v25);

smisbeh@0.68856 (v26);

tcollab@0.42719 (v27);

input@0.00075 (v28);

teff@0.69100 (v29);

[WJS] (js15);

[WOCC] (oc15);

%cg#1.c#6%

[ plbarrs@0.58396 ] (m7);

[ smisbeh@0.49068 ] (m8);

[ tcollab@-0.51802 ] (m9);

[ input@-0.66902 ] (m10);

[ teff@-0.41935 ] (m11);

plbarrs@0.63464 (v7);

smisbeh@0.87813 (v8);

tcollab@0.91497 (v9);

input@0.80201 (v10);

teff@0.76833 (v11);

[WJS] (js16);

[WOCC] (oc16);

*!Group 2 (England)*

%cg#2.c#1%

[ plbarrs@-0.44827 ] (m1);

[ smisbeh@-1.27932 ] (m2);

[ tcollab@0.22905 ] (m3);

[ input@0.36065 ] (m4);

[ teff@0.81401 ] (m5);

plbarrs@0.81505 (v1);

smisbeh@0.00059 (v2);

tcollab@0.52871 (v3);

input@0.77336 (v4);

teff@0.27371 (v5);

[WJS] (js21);

[WOCC] (oc21);

%cg#2.c#2%

[ plbarrs@-0.74323 ] (m13);

[ smisbeh@-0.16230 ] (m14);

[ tcollab@0.58975 ] (m15);

[ input@0.64659 ] (m16);

[ teff@0.46102 ] (m17);

plbarrs@0.64896 (v13);

smisbeh@0.47020 (v14);

tcollab@0.17542 (v15);

input@0.57663 (v16);

teff@0.48903 (v17);

[WJS] (js22);

[WOCC] (oc22);

%cg#2.c#3%

[ plbarrs@-0.29924 ] (m30);

[ smisbeh@-0.04876 ] (m31);

[ tcollab@0.44911 ] (m32);

[ input@0.37034 ] (m33);

[ teff@0.18890 ] (m34);

plbarrs@0.09013 (v30);

smisbeh@0.50253 (v31);

tcollab@0.19470 (v32);

input@0.00056 (v33);

teff@0.57300 (v34);

[WJS] (js23);

[WOCC] (oc23);

%cg#2.c#4%

[ plbarrs@0.09603 ] (m19);

[ smisbeh@-0.03531 ] (m20);

[ tcollab@-0.01181 ] (m21);

[ input@-0.08906 ] (m22);

[ teff@-0.09510 ] (m23);

plbarrs@0.52200 (v19);

smisbeh@0.00059 (v20);

tcollab@0.51633 (v21);

input@0.55427 (v22);

teff@0.32349 (v23);

[WJS] (js24);

[WOCC] (oc24);

%cg#2.c#5%

[ plbarrs@0.28596 ] (m25);

[ smisbeh@0.40055 ] (m26);

[ tcollab@-0.04868 ] (m27);

[ input@0.29653 ] (m28);

[ teff@-0.35182 ] (m29);

plbarrs@0.12338 (v25);

smisbeh@0.68856 (v26);

tcollab@0.42719 (v27);

input@0.00075 (v28);

teff@0.69100 (v29);

[WJS] (js25);

[WOCC] (oc25);

%cg#2.c#6%

[ plbarrs@0.58396 ] (m7);

[ smisbeh@0.49068 ] (m8);

[ tcollab@-0.51802 ] (m9);

[ input@-0.66902 ] (m10);

[ teff@-0.41935 ] (m11);

plbarrs@0.63464 (v7);

smisbeh@0.87813 (v8);

tcollab@0.91497 (v9);

input@0.80201 (v10);

teff@0.76833 (v11);

[WJS] (js26);

[WOCC] (oc26);

OUTPUT: STDYX CINTERVAL SVALUES RESIDUAL TECH1 TECH7;

**Mplus Input Syntax for L1 Profile Similarity Tests: Adjusted-Explanatory Similarity with Outcomes Constrained Across Countries While Controlling for Predictors (Step 2 of 2)**

DATA: FILE IS "STDWEIGHTS.dat";

VARIABLE: NAMES ARE PLBARRS SMISBEH TCOLLAB INPUT TEFF TT3G01 TT3G11B WOCC WJS BOCC BJS SCHLOC NENRSTUD TC3G17A TC3G17C BAUTSUPP BCOMPSUP BTENTH PV1MATHM PV2MATHM PV3MATHM PV4MATHM PV5MATHM PV6MATHM PV7MATHM PV8MATHM PV9MATHM PV10MATH PV1READM PV2READM PV3READM PV4READM PV5READM PV6READM PV7READM PV8READM PV9READM PV10READ PV1SCIEM PV2SCIEM PV3SCIEM PV4SCIEM PV5SCIEM PV6SCIEM PV7SCIEM PV8SCIEM PV9SCIEM PV10SCIE IDPOP TCHWGT SCHWGT TEACHID IDCNTRY SCHLID;

USEVARIABLES = PLBARRS SMISBEH TCOLLAB INPUT TEFF TT3G01 TT3G11B WOCC WJS;

KNOWNCLASS = cg (IDCNTRY = 36 IDCNTRY = 926);

CLASSES = cg (2) c (6);

CLUSTER = SCHLID;

MISSING ARE *;

WEIGHT =TCHWGT;

ANALYSIS:

TYPE = MIXTURE COMPLEX;

STARTS = 0;

MODEL:

%OVERALL%

C ON TT3G01 TT3G11B;

WOCC WJS ON TT3G01 TT3G11B;

*!Group 1 (Australia)*

%cg#1.c#1%

[ plbarrs@-0.44827 ] (m1);

[ smisbeh@-1.27932 ] (m2);

[ tcollab@0.22905 ] (m3);

[ input@0.36065 ] (m4);

[ teff@0.81401 ] (m5);

plbarrs@0.81505 (v1);

smisbeh@0.00059 (v2);

tcollab@0.52871 (v3);

input@0.77336 (v4);

teff@0.27371 (v5);

*!Parameter labels below refer to the mean levels of the outcome. In this syntax, the*

*! parameters are constrained to be equal for matching profiles across groups.*

[WJS] (js11);

[WOCC] (oc11);

%cg#1.c#2%

[ plbarrs@-0.74323 ] (m13);

[ smisbeh@-0.16230 ] (m14);

[ tcollab@0.58975 ] (m15);

[ input@0.64659 ] (m16);

[ teff@0.46102 ] (m17);

plbarrs@0.64896 (v13);

smisbeh@0.47020 (v14);

tcollab@0.17542 (v15);

input@0.57663 (v16);

teff@0.48903 (v17);

[WJS] (js12);

[WOCC] (oc12);

%cg#1.c#3%

[ plbarrs@-0.29924 ] (m30);

[ smisbeh@-0.04876 ] (m31);

[ tcollab@0.44911 ] (m32);

[ input@0.37034 ] (m33);

[ teff@0.18890 ] (m34);

plbarrs@0.09013 (v30);

smisbeh@0.50253 (v31);

tcollab@0.19470 (v32);

input@0.00056 (v33);

teff@0.57300 (v34);

[WJS] (js13);

[WOCC] (oc13);

%cg#1.c#4%

[ plbarrs@0.09603 ] (m19);

[ smisbeh@-0.03531 ] (m20);

[ tcollab@-0.01181 ] (m21);

[ input@-0.08906 ] (m22);

[ teff@-0.09510 ] (m23);

plbarrs@0.52200 (v19);

smisbeh@0.00059 (v20);

tcollab@0.51633 (v21);

input@0.55427 (v22);

teff@0.32349 (v23);

[WJS] (js14);

[WOCC] (oc14);

%cg#1.c#5%

[ plbarrs@0.28596 ] (m25);

[ smisbeh@0.40055 ] (m26);

[ tcollab@-0.04868 ] (m27);

[ input@0.29653 ] (m28);

[ teff@-0.35182 ] (m29);

plbarrs@0.12338 (v25);

smisbeh@0.68856 (v26);

tcollab@0.42719 (v27);

input@0.00075 (v28);

teff@0.69100 (v29);

[WJS] (js15);

[WOCC] (oc15);

%cg#1.c#6%

[ plbarrs@0.58396 ] (m7);

[ smisbeh@0.49068 ] (m8);

[ tcollab@-0.51802 ] (m9);

[ input@-0.66902 ] (m10);

[ teff@-0.41935 ] (m11);

plbarrs@0.63464 (v7);

smisbeh@0.87813 (v8);

tcollab@0.91497 (v9);

input@0.80201 (v10);

teff@0.76833 (v11);

[WJS] (js16);

[WOCC] (oc16);

*!Group 2 (England)*

%cg#2.c#1%

[ plbarrs@-0.44827 ] (m1);

[ smisbeh@-1.27932 ] (m2);

[ tcollab@0.22905 ] (m3);

[ input@0.36065 ] (m4);

[ teff@0.81401 ] (m5);

plbarrs@0.81505 (v1);

smisbeh@0.00059 (v2);

tcollab@0.52871 (v3);

input@0.77336 (v4);

teff@0.27371 (v5);

[WJS] (js11);

[WOCC] (oc11);

%cg#2.c#2%

[ plbarrs@-0.74323 ] (m13);

[ smisbeh@-0.16230 ] (m14);

[ tcollab@0.58975 ] (m15);

[ input@0.64659 ] (m16);

[ teff@0.46102 ] (m17);

plbarrs@0.64896 (v13);

smisbeh@0.47020 (v14);

tcollab@0.17542 (v15);

input@0.57663 (v16);

teff@0.48903 (v17);

[WJS] (js12);

[WOCC] (oc12);

%cg#2.c#3%

[ plbarrs@-0.29924 ] (m30);

[ smisbeh@-0.04876 ] (m31);

[ tcollab@0.44911 ] (m32);

[ input@0.37034 ] (m33);

[ teff@0.18890 ] (m34);

plbarrs@0.09013 (v30);

smisbeh@0.50253 (v31);

tcollab@0.19470 (v32);

input@0.00056 (v33);

teff@0.57300 (v34);

[WJS] (js13);

[WOCC] (oc13);

%cg#2.c#4%

[ plbarrs@0.09603 ] (m19);

[ smisbeh@-0.03531 ] (m20);

[ tcollab@-0.01181 ] (m21);

[ input@-0.08906 ] (m22);

[ teff@-0.09510 ] (m23);

plbarrs@0.52200 (v19);

smisbeh@0.00059 (v20);

tcollab@0.51633 (v21);

input@0.55427 (v22);

teff@0.32349 (v23);

[WJS] (js14);

[WOCC] (oc14);

%cg#2.c#5%

[ plbarrs@0.28596 ] (m25);

[ smisbeh@0.40055 ] (m26);

[ tcollab@-0.04868 ] (m27);

[ input@0.29653 ] (m28);

[ teff@-0.35182 ] (m29);

plbarrs@0.12338 (v25);

smisbeh@0.68856 (v26);

tcollab@0.42719 (v27);

input@0.00075 (v28);

teff@0.69100 (v29);

[WJS] (js15);

[WOCC] (oc15);

%cg#2.c#6%

[ plbarrs@0.58396 ] (m7);

[ smisbeh@0.49068 ] (m8);

[ tcollab@-0.51802 ] (m9);

[ input@-0.66902 ] (m10);

[ teff@-0.41935 ] (m11);

plbarrs@0.63464 (v7);

smisbeh@0.87813 (v8);

tcollab@0.91497 (v9);

input@0.80201 (v10);

teff@0.76833 (v11);

[WJS] (js16);

[WOCC] (oc16);

*!Model constraints required for tests of mean differences. One label (e.g. JS11v12) has to be*

*!created for each comparison using the NEW function.*

MODEL CONSTRAINT:

NEW (JS11v12 JS11v13 JS11v14 JS11v15 JS11v16 JS12v13 JS12v14 JS12v15 JS12v16 JS13v14 JS13v15 JS13v16 JS14v15 JS14v16 JS15v16 OC11v12 OC11v13 OC11v14 OC11v15 OC11v16 OC12v13 OC12v14 OC12v15 OC12v16 OC13v14 OC13v15 OC13v16 OC14v15 OC14v16 OC15v16);

*!Here are the tests used to compare outcome levels across the different profiles (there are no*

*! across group comparisons because matching profiles in each group/country were*

*! constrained to have equal outcome levels (i.e., explanatory similarity) in this syntax). The*

*! comparison is defined using the labels used for the outcomes in the main model (e.g, JS11).*

JS11v12 = js11-js12;

JS11v13 = js11-js13;

JS11v14 = js11-js14;

JS11v15 = js11-js15;

JS11v16 = js11-js16;

JS12v13 = js12-js13;

JS12v14 = js12-js14;

JS12v15 = js12-js15;

JS12v16 = js12-js16;

JS13v14 = js13-js14;

JS13v15 = js13-js15;

JS13v16 = js13-js16;

JS14v15 = js14-js15;

JS14v16 = js14-js16;

JS15v16 = js15-js16;

OC11v12 = oc11-oc12;

OC11v13 = oc11-oc13;

OC11v14 = oc11-oc14;

OC11v15 = oc11-oc15;

OC11v16 = oc11-oc16;

OC12v13 = oc12-oc13;

OC12v14 = oc12-oc14;

OC12v15 = oc12-oc15;

OC12v16 = oc12-oc16;

OC13v14 = oc13-oc14;

OC13v15 = oc13-oc15;

OC13v16 = oc13-oc16;

OC14v15 = oc14-oc15;

OC14v16 = oc14-oc16;

OC15v16 = oc15-oc16;

OUTPUT: STDYX CINTERVAL SVALUES RESIDUAL TECH1 TECH7;

**Mplus Input Syntax for L2 Profile Similarity Tests: L2 Predictive Similarity with Associations Between Predictors and the Likelihood of Profile Membership Freely Estimated Across Countries (Step 1 of 2)**

DATA: FILE IS "GLOBALDAT.csv";

VARIABLE: NAMES ARE PLBARRS SMISBEH TCOLLAB INPUT TEFF TT3G01 TCHAGEGR TT3G11B TT3G03 WOCC WJS BOCC BJS TC3G01 PRAGEGR TC3G03 TC3G04B SCHLOC TC3G12 NENRSTUD TC3G17A TC3G17B TC3G17C TC3G17D TC3G17E BAUTSUPP BCOMPSUP BTENTH PV1MATHM PV2MATHM PV3MATHM PV4MATHM PV5MATHM PV6MATHM PV7MATHM PV8MATHM PV9MATHM PV10MATH PV1READM PV2READM PV3READM PV4READM PV5READM PV6READM PV7READM PV8READM PV9READM PV10READ PV1SCIEM PV2SCIEM PV3SCIEM PV4SCIEM PV5SCIEM PV6SCIEM PV7SCIEM PV8SCIEM PV9SCIEM PV10SCIE ESCSM IDPOP IDCNTRY SCHWGT CPROB1 CPROB2 CPROB3 CPROB4 CPROB5 CPROB6 CL TCHWGT TEACHID SCHLID;

USEVARIABLES = CL SCHLOC NENRSTUD TC3G17C TC3G17A;

NOMINAL = CL;

KNOWNCLASS = cg (IDCNTRY = 36 IDCNTRY = 926);

CLASSES = cg (2) cb (2) cw (6);

WITHIN = CL;

BETWEEN = CG CB SCHLOC NENRSTUD TC3G17C TC3G17A;

CLUSTER = SCHLID;

MISSING ARE *;

weight=TCHWGT;

wtscale = cluster;

bweight=SCHWGT;

bwtscale = sample;

ANALYSIS:

TYPE = MIXTURE TWOLEVEL;

STARTS = 0;

Algorithm = Integration;

Integration = Montecarlo;

MODEL:

*!The start values below are taken from the output of the most constrained similarity test !(SVALUES). In our case, that was the L2-Distributional similarity test.*

%WITHIN%

%OVERALL%

[ cw#1@-2.14045 ];

[ cw#2@-1.43284 ];

[ cw#3@-1.62790 ];

[ cw#4@-1.35196 ];

[ cw#5@-1.28622 ];

%BETWEEN%

%OVERALL%

cw#1 ON cb#1@1.75615;

cw#2 ON cb#1@1.52476;

cw#3 ON cb#1@0.88255;

cw#4 ON cb#1@1.06918;

cw#5 ON cb#1@0.19555;

*!The association between predictors and the L2 latent profiles needs to be constrained to zero*

*!below in the overall statement in order for the model to be identified and allow testing of this*

*!association separately in each separate group (i.e., CG).*

CB ON SCHLOC@0 NENRSTUD@0 TC3G17C@0 TC3G17A@0;

*!By* *requesting the free estimation of the variance of the predictors here, it will prevent any*

*!cases with missing data on the exogenous predictors from being deleted from analyses. To*

*! allow for this (to implement full information maximum likelihood for exogenous predictors*

*!in mixture models), it is necessary to include “ALGORITHM = INTEGRATION;” and*

*!“INTEGRATION = MONTECARLO;” in the ANALYSIS section (above).*

SCHLOC NENRSTUD TC3G17C TC3G17A;

MODEL CG.CW:

%WITHIN%

*!The start values below are those from the third step of the Manual 3-Step Process. Details
!about obtaining these values are described in Collie et al. (2020).*

*!Group1 (Australia)*

%cg#1.cw#1%

[CL#1@4.102];

[CL#2@-1.081];

[CL#3@-0.507];

[CL#4@-9.679];

[CL#5@-1.553];

%cg#1.cw#2%

[CL#1@-1.318];

[CL#2@2.213];

[CL#3@-1.104];

[CL#4@-0.825];

[CL#5@-2.221];

%cg#1.cw#3%

[CL#1@1.691];

[CL#2@0.972];

[CL#3@5.133];

[CL#4@1.582];

[CL#5@2.04];

%cg#1.cw#4%

[CL#1@-10.38];

[CL#2@-0.07];

[CL#3@0.441];

[CL#4@3.278];

[CL#5@0.038];

%cg#1.cw#5%

[CL#1@-0.883];

[CL#2@-1.536];

[CL#3@1.063];

[CL#4@1.165];

[CL#5@3.808];

%cg#1.cw#6%

[CL#1@-4.916];

[CL#2@-3.048];

[CL#3@-5.163];

[CL#4@-3.46];

[CL#5@-3.834];

*!Group 2 (England)*

%cg#2.cw#1%

[CL#1@4.074];

[CL#2@-1.6];

[CL#3@-0.637];

[CL#4@-9.708];

[CL#5@-1.3];

%cg#2.cw#2%

[CL#1@-1.41];

[CL#2@1.953];

[CL#3@-1.082];

[CL#4@-1.062];

[CL#5@-1.719];

%cg#2.cw#3%

[CL#1@2.868];

[CL#2@1.546];

[CL#3@5.915];

[CL#4@2.267];

[CL#5@3.027];

%cg#2.cw#4%

[CL#1@-10.618];

[CL#2@-0.609];

[CL#3@-0.125];

[CL#4@3.047];

[CL#5@0.009];

%cg#2.cw#5%

[CL#1@-0.414];

[CL#2@-2.395];

[CL#3@0.717];

[CL#4@1.181];

[CL#5@3.859];

%cg#2.cw#6%

[CL#1@-4.774];

[CL#2@-3.401];

[CL#3@-5.752];

[CL#4@-3.418];

[CL#5@-3.829];

*!Below is where the predictors are regressed on the between-level latent variable (CB)
!separately for each country.*

MODEL CG:

%BETWEEN%

*!Group 1 (Australia)*

%cg#1%

cb#1 ON SCHLOC NENRSTUD TC3G17C TC3G17A;

*!Group 2 (England)*

%cg#2%

cb#1 ON SCHLOC NENRSTUD TC3G17C TC3G17A;

OUTPUT: STDYX CINTERVAL SVALUES RESIDUAL TECH1 TECH7;

**Mplus Input Syntax for L2 Profile Similarity Tests: L2 Predictive Similarity with Associations Between Predictors and the Likelihood of Profile Membership Constrained Across Countries (Step 2 of 2)**

DATA: FILE IS "GLOBALDAT.csv";

VARIABLE: NAMES ARE PLBARRS SMISBEH TCOLLAB INPUT TEFF TT3G01 TCHAGEGR TT3G11B TT3G03 WOCC WJS BOCC BJS TC3G01 PRAGEGR TC3G03 TC3G04B SCHLOC TC3G12 NENRSTUD TC3G17A TC3G17B TC3G17C TC3G17D TC3G17E BAUTSUPP BCOMPSUP BTENTH PV1MATHM PV2MATHM PV3MATHM PV4MATHM PV5MATHM PV6MATHM PV7MATHM PV8MATHM PV9MATHM PV10MATH PV1READM PV2READM PV3READM PV4READM PV5READM PV6READM PV7READM PV8READM PV9READM PV10READ PV1SCIEM PV2SCIEM PV3SCIEM PV4SCIEM PV5SCIEM PV6SCIEM PV7SCIEM PV8SCIEM PV9SCIEM PV10SCIE ESCSM IDPOP IDCNTRY SCHWGT CPROB1 CPROB2 CPROB3 CPROB4 CPROB5 CPROB6 CL TCHWGT TEACHID SCHLID;

USEVARIABLES = CL SCHLOC NENRSTUD TC3G17C TC3G17A;

NOMINAL = CL;

KNOWNCLASS = cg (IDCNTRY = 36 IDCNTRY = 926);

CLASSES = cg (2) cb (2) cw (6);

WITHIN = CL;

BETWEEN = CG CB SCHLOC NENRSTUD TC3G17C TC3G17A;

CLUSTER = SCHLID;

MISSING ARE *;

weight=TCHWGT;

wtscale = cluster;

bweight=SCHWGT;

bwtscale = sample;

ANALYSIS:

TYPE = MIXTURE TWOLEVEL;

STARTS = 0;

Algorithm = Integration;

Integration = Montecarlo;

MODEL:

%WITHIN%

%OVERALL%

[ cw#1@-2.14045 ];

[ cw#2@-1.43284 ];

[ cw#3@-1.62790 ];

[ cw#4@-1.35196 ];

[ cw#5@-1.28622 ];

%BETWEEN%

%OVERALL%

cw#1 ON cb#1@1.75615;

cw#2 ON cb#1@1.52476;

cw#3 ON cb#1@0.88255;

cw#4 ON cb#1@1.06918;

cw#5 ON cb#1@0.19555;

*!The associations between predictors and the between-level profiles (CB) are now estimated*

*!in the overall statement to allow the associations to be constrained across groups (i.e., CG).*

CB ON SCHLOC NENRSTUD TC3G17C TC3G17A;

SCHLOC NENRSTUD TC3G17C TC3G17A;

MODEL CG.CW:

%WITHIN%

*!Group1 (Australia)*

%cg#1.cw#1%

[CL#1@4.102];

[CL#2@-1.081];

[CL#3@-0.507];

[CL#4@-9.679];

[CL#5@-1.553];

%cg#1.cw#2%

[CL#1@-1.318];

[CL#2@2.213];

[CL#3@-1.104];

[CL#4@-0.825];

[CL#5@-2.221];

%cg#1.cw#3%

[CL#1@1.691];

[CL#2@0.972];

[CL#3@5.133];

[CL#4@1.582];

[CL#5@2.04];

%cg#1.cw#4%

[CL#1@-10.38];

[CL#2@-0.07];

[CL#3@0.441];

[CL#4@3.278];

[CL#5@0.038];

%cg#1.cw#5%

[CL#1@-0.883];

[CL#2@-1.536];

[CL#3@1.063];

[CL#4@1.165];

[CL#5@3.808];

%cg#1.cw#6%

[CL#1@-4.916];

[CL#2@-3.048];

[CL#3@-5.163];

[CL#4@-3.46];

[CL#5@-3.834];

*!Group 2 (England)*

%cg#2.cw#1%

[CL#1@4.074];

[CL#2@-1.6];

[CL#3@-0.637];

[CL#4@-9.708];

[CL#5@-1.3];

%cg#2.cw#2%

[CL#1@-1.41];

[CL#2@1.953];

[CL#3@-1.082];

[CL#4@-1.062];

[CL#5@-1.719];

%cg#2.cw#3%

[CL#1@2.868];

[CL#2@1.546];

[CL#3@5.915];

[CL#4@2.267];

[CL#5@3.027];

%cg#2.cw#4%

[CL#1@-10.618];

[CL#2@-0.609];

[CL#3@-0.125];

[CL#4@3.047];

[CL#5@0.009];

%cg#2.cw#5%

[CL#1@-0.414];

[CL#2@-2.395];

[CL#3@0.717];

[CL#4@1.181];

[CL#5@3.859];

%cg#2.cw#6%

[CL#1@-4.774];

[CL#2@-3.401];

[CL#3@-5.752];

[CL#4@-3.418];

[CL#5@-3.829];

OUTPUT: STDYX CINTERVAL SVALUES RESIDUAL TECH1 TECH7;

**Mplus Syntax for L2 Profile Similarity Tests: L2 Adjusted-Explanatory Similarity with Outcomes Freely Estimated Across Countries While Controlling for Predictors (Step 1 of 2)**

DATA: FILE IS "GLOBALDAT.csv";

VARIABLE: NAMES ARE PLBARRS SMISBEH TCOLLAB INPUT TEFF TT3G01 TCHAGEGR TT3G11B TT3G03 WOCC WJS BOCC BJS TC3G01 PRAGEGR TC3G03 TC3G04B SCHLOC TC3G12 NENRSTUD TC3G17A TC3G17B TC3G17C TC3G17D TC3G17E BAUTSUPP BCOMPSUP BTENTH PV1MATHM PV2MATHM PV3MATHM PV4MATHM PV5MATHM PV6MATHM PV7MATHM PV8MATHM PV9MATHM PV10MATH PV1READM PV2READM PV3READM PV4READM PV5READM PV6READM PV7READM PV8READM PV9READM PV10READ PV1SCIEM PV2SCIEM PV3SCIEM PV4SCIEM PV5SCIEM PV6SCIEM PV7SCIEM PV8SCIEM PV9SCIEM PV10SCIE ESCSM IDPOP IDCNTRY SCHWGT CPROB1 CPROB2 CPROB3 CPROB4 CPROB5 CPROB6 CL TCHWGT TEACHID SCHLID;

USEVARIABLES = CL SCHLOC NENRSTUD TC3G17C TC3G17A BJSm BOCCm;

NOMINAL = CL;

KNOWNCLASS = cg (IDCNTRY = 36 IDCNTRY = 926);

CLASSES = cg (2) cb (2) cw (6);

WITHIN = CL;

BETWEEN = CG CB SCHLOC NENRSTUD TC3G17C TC3G17A BJSm BOCCm;

CLUSTER = SCHLID;

MISSING ARE *;

weight=TCHWGT;

wtscale = cluster;

bweight=SCHWGT;

bwtscale = sample;

DEFINE:

*!The cluster means were created to account for the fact that some individual teachers had*

*! missing data on the factor scores for the outcomes that were created and merged in the*

*!separate tests of measurement invariance. This meant there was within-cluster variance due*

*!to missing data (so, false within-cluster variance). The cluster_means solve this issue by*

*!making sure that all teachers from the same school have the same school level score.*

BJSm=cluster_mean(BJS);

BOCCm=cluster_mean(BOCC);

ANALYSIS:

TYPE = MIXTURE TWOLEVEL;

STARTS = 0;

Algorithm = Integration;

Integration = Montecarlo;

MODEL:

%WITHIN%

%OVERALL%

[ cw#1@-2.14045 ];

[ cw#2@-1.43284 ];

[ cw#3@-1.62790 ];

[ cw#4@-1.35196 ];

[ cw#5@-1.28622 ];

%BETWEEN%

%OVERALL%

cw#1 ON cb#1@1.75615;

cw#2 ON cb#1@1.52476;

cw#3 ON cb#1@0.88255;

cw#4 ON cb#1@1.06918;

cw#5 ON cb#1@0.19555;

*!Below, the predictors are regressed on the latent profile variable (CB) and the outcomes.*

*!For the associations between the predictors and the latent profile variable (CB), this means !the associations were constrained to be equal across groups. If we had not attained L2-*

*!Predictive similarity, these associations would need to be freely estimated across groups*

CB ON SCHLOC NENRSTUD TC3G17C TC3G17A;

BJSm BOCCm ON SCHLOC NENRSTUD TC3G17C TC3G17A;

SCHLOC NENRSTUD TC3G17C TC3G17A;

MODEL CG.CW:

%WITHIN%

*!Group 1 (Australia)*

%cg#1.cw#1%

[CL#1@4.102];

[CL#2@-1.081];

[CL#3@-0.507];

[CL#4@-9.679];

[CL#5@-1.553];

%cg#1.cw#2%

[CL#1@-1.318];

[CL#2@2.213];

[CL#3@-1.104];

[CL#4@-0.825];

[CL#5@-2.221];

%cg#1.cw#3%

[CL#1@1.691];

[CL#2@0.972];

[CL#3@5.133];

[CL#4@1.582];

[CL#5@2.04];

%cg#1.cw#4%

[CL#1@-10.38];

[CL#2@-0.07];

[CL#3@0.441];

[CL#4@3.278];

[CL#5@0.038];

%cg#1.cw#5%

[CL#1@-0.883];

[CL#2@-1.536];

[CL#3@1.063];

[CL#4@1.165];

[CL#5@3.808];

%cg#1.cw#6%

[CL#1@-4.916];

[CL#2@-3.048];

[CL#3@-5.163];

[CL#4@-3.46];

[CL#5@-3.834];

*!Group 2 (England)*

%cg#2.cw#1%

[CL#1@4.074];

[CL#2@-1.6];

[CL#3@-0.637];

[CL#4@-9.708];

[CL#5@-1.3];

%cg#2.cw#2%

[CL#1@-1.41];

[CL#2@1.953];

[CL#3@-1.082];

[CL#4@-1.062];

[CL#5@-1.719];

%cg#2.cw#3%

[CL#1@2.868];

[CL#2@1.546];

[CL#3@5.915];

[CL#4@2.267];

[CL#5@3.027];

%cg#2.cw#4%

[CL#1@-10.618];

[CL#2@-0.609];

[CL#3@-0.125];

[CL#4@3.047];

[CL#5@0.009];

%cg#2.cw#5%

[CL#1@-0.414];

[CL#2@-2.395];

[CL#3@0.717];

[CL#4@1.181];

[CL#5@3.859];

%cg#2.cw#6%

[CL#1@-4.774];

[CL#2@-3.401];

[CL#3@-5.752];

[CL#4@-3.418];

[CL#5@-3.829];

*!Below is where differences in the outcomes at the school-level are tested across groups (i.e.,*

*!the two countries)*

MODEL CG.CB:

%BETWEEN%

*!Group 1 (Australia)*

%cg#1.cb#1%

[BJSm] (js1aus);

[BOCCm] (oc1aus);

%cg#1.cb#2%

[BJSm] (js2aus);

[BOCCm] (oc2aus);

*!Group 2 (England)*

%cg#2.cb#1%

[BJSm] (js1eng);

[BOCCm] (oc1eng);

%cg#2.cb#2%

[BJSm] (js2eng);

[BOCCm] (oc2eng);

*!Model constraints allow for tests of mean differences. The results of the tests are controlling !for the effects of the predictors*

MODEL CONSTRAINT:

NEW (jobaus12 occaus12 jobeng12 occeng12 job1cc occ1cc job2cc occ2cc);

*!Comparisons across profiles in Australia*

jobaus12 = js1aus-js2aus;

occaus12 = oc1aus-oc2aus;

*!Comparisons across profiles in England*

jobeng12 = js1eng-js2eng;

occeng12 = oc1eng-oc2eng;

*!Comparisons across profiles across country (comparing like L2 profiles across Australia and England)*

job1cc = js1aus-js1eng;

occ1cc = oc1aus-oc1eng;

job2cc = js2aus-js2eng;

occ2cc = oc2aus-oc2eng;

OUTPUT: STDYX CINTERVAL SVALUES RESIDUAL TECH1 TECH7;

**Mplus Input Syntax for L2 Profile Similarity Tests: L2 Adjusted-Explanatory Similarity with Outcomes Constrained Across Countries While Controlling for Predictors (Step 2 of 2)**

DATA: FILE IS "GLOBALDAT.csv";

VARIABLE: NAMES ARE PLBARRS SMISBEH TCOLLAB INPUT TEFF TT3G01 TCHAGEGR TT3G11B TT3G03 WOCC WJS BOCC BJS TC3G01 PRAGEGR TC3G03 TC3G04B SCHLOC TC3G12 NENRSTUD TC3G17A TC3G17B TC3G17C TC3G17D TC3G17E BAUTSUPP BCOMPSUP BTENTH PV1MATHM PV2MATHM PV3MATHM PV4MATHM PV5MATHM PV6MATHM PV7MATHM PV8MATHM PV9MATHM PV10MATH PV1READM PV2READM PV3READM PV4READM PV5READM PV6READM PV7READM PV8READM PV9READM PV10READ PV1SCIEM PV2SCIEM PV3SCIEM PV4SCIEM PV5SCIEM PV6SCIEM PV7SCIEM PV8SCIEM PV9SCIEM PV10SCIE ESCSM IDPOP IDCNTRY SCHWGT CPROB1 CPROB2 CPROB3 CPROB4 CPROB5 CPROB6 CL TCHWGT TEACHID SCHLID;

USEVARIABLES = CL SCHLOC NENRSTUD TC3G17C TC3G17A BJSm BOCCm;

NOMINAL = CL;

KNOWNCLASS = cg (IDCNTRY = 36 IDCNTRY = 926);

CLASSES = cg (2) cb (2) cw (6);

WITHIN = CL;

BETWEEN = CG CB SCHLOC NENRSTUD TC3G17C TC3G17A BJSm BOCCm;

CLUSTER = SCHLID;

MISSING ARE *;

weight=TCHWGT;

wtscale = cluster;

bweight=SCHWGT;

bwtscale = sample;

DEFINE:

BJSm=cluster_mean(BJS);

BOCCm=cluster_mean(BOCC);

ANALYSIS:

TYPE = MIXTURE TWOLEVEL;

STARTS = 0;

Algorithm = Integration;

Integration = Montecarlo;

MODEL:

%WITHIN%

%OVERALL%

[ cw#1@-2.14045 ];

[ cw#2@-1.43284 ];

[ cw#3@-1.62790 ];

[ cw#4@-1.35196 ];

[ cw#5@-1.28622 ];

%BETWEEN%

%OVERALL%

cw#1 ON cb#1@1.75615;

cw#2 ON cb#1@1.52476;

cw#3 ON cb#1@0.88255;

cw#4 ON cb#1@1.06918;

cw#5 ON cb#1@0.19555;

CB ON SCHLOC NENRSTUD TC3G17C TC3G17A;

BJSm BOCCm ON SCHLOC NENRSTUD TC3G17C TC3G17A;

SCHLOC NENRSTUD TC3G17C TC3G17A;

MODEL CG.CW:

%WITHIN%

*!Group 1 (Australia)*

%cg#1.cw#1%

[CL#1@4.102];

[CL#2@-1.081];

[CL#3@-0.507];

[CL#4@-9.679];

[CL#5@-1.553];

%cg#1.cw#2%

[CL#1@-1.318];

[CL#2@2.213];

[CL#3@-1.104];

[CL#4@-0.825];

[CL#5@-2.221];

%cg#1.cw#3%

[CL#1@1.691];

[CL#2@0.972];

[CL#3@5.133];

[CL#4@1.582];

[CL#5@2.04];

%cg#1.cw#4%

[CL#1@-10.38];

[CL#2@-0.07];

[CL#3@0.441];

[CL#4@3.278];

[CL#5@0.038];

%cg#1.cw#5%

[CL#1@-0.883];

[CL#2@-1.536];

[CL#3@1.063];

[CL#4@1.165];

[CL#5@3.808];

%cg#1.cw#6%

[CL#1@-4.916];

[CL#2@-3.048];

[CL#3@-5.163];

[CL#4@-3.46];

[CL#5@-3.834];

*!Group 2 (England)*

%cg#2.cw#1%

[CL#1@4.074];

[CL#2@-1.6];

[CL#3@-0.637];

[CL#4@-9.708];

[CL#5@-1.3];

%cg#2.cw#2%

[CL#1@-1.41];

[CL#2@1.953];

[CL#3@-1.082];

[CL#4@-1.062];

[CL#5@-1.719];

%cg#2.cw#3%

[CL#1@2.868];

[CL#2@1.546];

[CL#3@5.915];

[CL#4@2.267];

[CL#5@3.027];

%cg#2.cw#4%

[CL#1@-10.618];

[CL#2@-0.609];

[CL#3@-0.125];

[CL#4@3.047];

[CL#5@0.009];

%cg#2.cw#5%

[CL#1@-0.414];

[CL#2@-2.395];

[CL#3@0.717];

[CL#4@1.181];

[CL#5@3.859];

%cg#2.cw#6%

[CL#1@-4.774];

[CL#2@-3.401];

[CL#3@-5.752];

[CL#4@-3.418];

[CL#5@-3.829];

*!Below, the means of the outcomes are constrained to equality across group (i.e., the two*

*!countries)*

MODEL CG.CB:

%BETWEEN%

*!Group 1 (Australia)*

%cg#1.cb#1%

[BJSm] (js1);

[BOCCm] (oc1);

%cg#1.cb#2%

[BJSm] (js2);

[BOCCm] (oc2);

*!Group 2 (England)*

%cg#2.cb#1%

[BJSm] (js1);

[BOCCm] (oc1);

%cg#2.cb#2%

[BJSm] (js2);

[BOCCm] (oc2);

OUTPUT: STDYX CINTERVAL SVALUES RESIDUAL TECH1 TECH7;
